# The Plant Growth Regulator Forchlorfenuron (KT-30) Drives Atherosclerosis Progression via Lipid Homeostasis Disruption: Evidence from ApoE-Deficient Mice

**DOI:** 10.3390/antiox15080953

**Published:** 2026-07-30

**Authors:** Chia-Hui Chen, Po-An Hu, Chun-Sheng Chuang, Wen-Hua Chen, Hua-Yu Tang, Chiao-Po Hsu, Tzong-Shyuan Lee

**Affiliations:** 1 Graduate Institute and Department of Physiology, College of Medicine, National Taiwan University, Taipei 10051, Taiwan; chiahuichen1993@ntu.edu.tw (C.-H.C.); d07441003@ntu.edu.tw (P.-A.H.); d13441003@ntu.edu.tw (C.-S.C.); f08441005@ntu.edu.tw (W.-H.C.); r14441005@ntu.edu.tw (H.-Y.T.); 2 Division of Cardiovascular Surgery, Department of Surgery, Taipei Veterans General Hospital, Taipei 11221, Taiwan

**Keywords:** forchlorfenuron, KT-30, atherosclerosis, oxidative stress, NADPH oxidase, dyslipidemia, hepatic steatosis

## Abstract

Oxidative stress is a central driver of atherosclerosis progression, promoting lipid peroxidation, vascular inflammation, and hepatic metabolic dysfunction. Forchlorfenuron (KT-30), a cytokinin-like plant growth regulator widely used on fruits such as kiwifruit, grapes, and watermelon, has been reported to elevate serum cholesterol levels, suggesting potential atherogenic effects. However, whether KT-30 induces oxidative stress and accelerates atherosclerosis remains unknown. Here, apolipoprotein E-deficient (apoE^−/−^) mice were orally administered KT-30 (5 mg/kg/day) for four weeks. KT-30 significantly accelerated atherosclerotic lesion formation, elevated plasma cholesterol levels, upregulated scavenger receptors SR-A and CD36, and downregulated ABCA1 and LXRα, indicating impaired reverse cholesterol transport and enhanced foam cell formation. KT-30 also increased pro-inflammatory cytokines (IL-1β, IL-6, MIP-2) and aortic expression of F4/80 and VCAM-1. Critically, KT-30 exposure was associated with elevated oxidative stress markers, as evidenced by elevated plasma MDA levels, increased aortic 4-HNE immunostaining, and upregulation of NOX-1/4. In the liver, KT-30 induced lipid accumulation, characterized by elevated cholesterol and free fatty acids, accompanied by SREBP-1/2-driven de novo lipogenesis and impaired lipoprotein uptake. Proteomic analysis revealed significant alterations in mitochondrial oxidative phosphorylation- and sirtuin signaling-related protein expression. Taken together, KT-30-associated oxidative stress, accompanied by upregulation of NOX-1/4 and alterations in mitochondrial pathway-related protein expression, may represent an important mechanistic link between lipid homeostasis disruption and accelerated atherosclerosis in apoE^−/−^ mice, highlighting the potential pro-atherogenic effects of KT-30 in a susceptible experimental model and providing mechanistic evidence that warrants further investigation of its possible cardiovascular implications.

## 1. Introduction

Atherosclerosis is a chronic inflammatory disease of the arterial wall and remains one of the most prevalent cardiovascular disorders in developed countries [1,2]. Its development is strongly associated with metabolic syndrome, which encompasses obesity, dyslipidemia, hypertension, diabetes mellitus, and hyperuricemia, conditions that collectively accelerate vascular dysfunction and plaque formation [1,3,4,5]. Elevated circulating cholesterol is a central initiating factor in atherogenesis [6]. In particular, excess low-density lipoprotein (LDL) undergoes oxidative modification to form oxidized LDL (oxLDL), which promotes endothelial dysfunction and triggers a local inflammatory cascade that attracts circulating monocytes [7]. Following transmigration into the subendothelial space, these monocytes differentiate into macrophages and take up oxLDL primarily through receptors such as cluster of differentiation 36 (CD36) and class A scavenger receptor (SR-A). Progressive lipid uptake transforms macrophages into foam cells, driving plaque growth and contributing to the formation of unstable atherosclerotic lesions [8,9,10]. With continued accumulation, these lesions may rupture, leading to thrombosis and acute cardiovascular events [11,12]. Among the various mechanisms driving atherogenesis, oxidative stress has emerged as a central and unifying pathological feature. Excess reactive oxygen species (ROS) promote oxidative modification of LDL, impair endothelial function, activate pro-inflammatory signaling cascades, and disrupt vascular redox homeostasis, collectively accelerating plaque formation and progression [10,11,12].

Beyond its role in vascular pathology, dyslipidemia also reflects disturbances in hepatic lipid metabolism [3,13]. As the central organ responsible for lipid homeostasis, the liver regulates multiple pathways, including de novo lipogenesis, triglyceride synthesis, fatty acid uptake, and lipoprotein secretion and clearance, to maintain systemic lipid balance [14,15,16]. De novo lipogenesis, controlled largely by sterol regulatory element-binding proteins 1 and 2 (SREBP-1/2), plays a crucial role in endogenous lipid production and is closely linked to the development of dyslipidemia [15,17]. Consequently, lifestyle modifications, such as increased physical activity and greater consumption of fruits and vegetables, are widely recommended to promote metabolic health. Importantly, hepatic oxidative stress plays a pivotal role in amplifying lipid metabolic dysfunction. Excessive ROS generation impairs mitochondrial β-oxidation, activates SREBP-driven de novo lipogenesis, and promotes lipid peroxidation, thereby establishing a feed-forward cycle that worsens both hepatic steatosis and systemic dyslipidemia [12,15].

To enhance fruit size and market value, the plant growth regulator KT-30 (forchlorfenuron) is commonly applied in agricultural production. KT-30 is used in a wide range of fruits, including kiwifruit, grapes, and watermelons [18,19,20]. Its effectiveness in improving fruit quality has led to increasingly widespread use, prompting investigation into the potential impacts on human health through dietary exposure. Although several toxicological studies reported that KT-30 does not significantly alter body weight, organ mass, or histological features in rodents [21,22], other evidence suggests potential metabolic effects. Notably, the Australian Pesticides and Veterinary Medicines Authority reported that high-dose KT-30 exposure increased serum cholesterol levels in dogs [23]. Given the established role of elevated cholesterol in promoting atherosclerosis [24], these observations suggest that KT-30 may disrupt lipid metabolism and exacerbate atherogenesis. However, its effects on atherosclerosis development and lipid homeostasis have not been systematically investigated. Notably, the potential of KT-30 to induce oxidative stress and whether such an oxidative insult may serve as a mechanistic bridge between lipid metabolic disruption and accelerated atherosclerosis have not been investigated. Given the established role of ROS in atherogenesis and hepatic lipid dysregulation, we hypothesized that oxidative stress may contribute to KT-30-associated metabolic and vascular alterations, which in turn disrupt vascular and hepatic lipid homeostasis. As oxidative stress reflects an imbalance between pro-oxidant generation and endogenous antioxidant defense capacity, elucidating whether KT-30 disrupts this balance, through excessive ROS production, impaired antioxidant enzyme regulation, or both, is essential to understanding its cardiovascular and metabolic consequences.

The present study is also situated within a broader and growing body of evidence linking exposure to agrochemicals and environmental chemicals to cardiovascular toxicity through oxidative stress-related mechanisms. Systematic reviews of occupational and environmental pesticide exposure have consistently reported associations with increased cardiovascular disease risk, including hypertension and atherosclerosis, with oxidative stress and endothelial dysfunction identified as recurring mechanistic themes [25,26]. Mechanistic animal studies further support a causal contribution of pesticide-related oxidative injury to atherogenesis: mice deficient in serum paraoxonase, an enzyme responsible for detoxifying organophosphate compounds and protecting lipoproteins from oxidative modification, exhibit heightened susceptibility to both organophosphate toxicity and atherosclerosis [9,27,28]. Similarly, NADPH oxidase isoforms, including NOX1, have been directly implicated in lesion development in apoE^−/−^ mice [29], paralleling the upregulation of NOX1 and NOX4 observed in the present study. Beyond classical pesticides, other agriculturally or industrially derived chemicals with dietary exposure routes, such as bisphenol A and di-(2-ethylhexyl) phthalate, have also been shown to accelerate atherosclerotic lesion formation in apoE-deficient mouse models [9,28], illustrating a broader pattern in which non-nutritive agrochemical and industrial compounds can promote atherogenesis via oxidative and lipid-homeostatic disruption. These converging lines of evidence support the biological plausibility and relevance of investigating KT-30, a structurally distinct but similarly xenobiotic plant growth regulator, as a potential dietary contributor to oxidative stress-driven cardiovascular risk.

To address this gap, the present study examines whether KT-30 accelerates atherosclerotic lesion formation using apolipoprotein E knockout (apoE^−/−^) mice, a well-established animal model of spontaneous hypercholesterolemia and atherosclerosis. Furthermore, we assess KT-30-induced oxidative stress responses, including ROS generation, lipid peroxidation, and NADPH oxidase activation, alongside changes in hepatic and vascular lipid metabolism and inflammatory signaling, to elucidate the molecular pathways through which KT-30-driven oxidative injury may contribute to dyslipidemia and accelerate atherosclerosis progression. We propose that oxidative stress represents the central mechanistic hub linking KT-30 exposure to its downstream cardiovascular and metabolic consequences.

## 2. Materials and Methods

### 2.1. Reagents and Antibodies

KT-30 was from Cayman Chemical (Ann Arbor, MI, USA). Rabbit antibodies for ATP binding cassette G1 (ABCG1, ab52617), ATP binding cassette G8 (ABCG8, ab223056), acyl-CoA oxidase 1 (ACOX-1, ab59964), CD36 (ab133625), carnitine palmitoyltransferase 1A (CPT1A, ab128568), intercellular adhesion molecule 1 (ICAM-1, ab124759), low-density lipoprotein receptor (LDLR, ab52818), low-density lipoprotein receptor-related protein 1 (LRP1, ab92544), peroxisome proliferator-activated receptor gamma coactivator 1-alpha (PGC-1α, ab54481), scavenger receptor class B type 1 (SR-BI, ab52629) and vascular cell adhesion molecule 1 (VCAM-1, ab134047), mouse antibodies for liver X receptor alpha (LXRα, ab4902) and ATP binding cassette A1 (ABCA1, ab18180), rat antibody for F4/80 (ab6640) were from Abcam (Cambridge, UK). Goat antibodies for acetyl CoA acetyltransferase 1 (ACAT1, sc-161307), microsomal triglyceride transfer protein (MTP, sc-33116) and SR-A (sc-20444), the rabbit antibodies for long-chain-fatty-acid—CoA ligase 1 (ACSL1, sc-98925), ATP binding cassette G5 (ABCG5, sc-25796), diglyceride acyltransferase 1 (DGAT1, sc-32861), inducible nitric oxide synthase (iNOS, sc-651) and liver fatty acid-binding protein (L-FABP, sc50380), mouse antibodies for cytochrome P450 family 7 subfamily A member 1 (CYP7A1, sc-518007) and 3-hydroxy-3-methyl-glutaryl-coenzyme A reductase (HMGCR, sc-271595) were from Santa Cruz Biotechnology (Santa Cruz, CA, USA). Mouse antibodies for SREBP-1 (557036) and SREBP-2 (557037) were from BD Biosciences (Franklin Lakes, NJ, USA). Goat anti-diglyceride acyltransferase 2 (DGAT2; NB100-57851) antibody was purchased from Novus Biologicals (Centennial, CO, USA). Rabbit antibody for adipose triglyceride lipase (ATGL, #2138) was obtained from Cell Signaling Technology (Beverly, MA, USA). Rabbit antibodies for apolipoprotein AI (apo-AI, 14427-1-AP), apolipoprotein B (apoB, 20578-1-AP), NADPH oxidase 1 (NOX-1, 17772-1-AP), NADPH oxidase 4 (NOX-4, 14347-1-AP), peroxisome proliferator-activated receptor alpha (PPARα, 15540-1-AP), and goat antibody for acetyl CoA acetyltransferase 2 (ACAT2, 14755-1-AP) were purchased from Proteintech (Rosemont, IL, USA). Rabbit anti-lysosomal acid lipase (LAL; GTX101169) antibody was purchased from GeneTex (Irvine, CA, USA). The mouse anti-β-actin antibody (AC004) was purchased from Abclonal (Woburn, MA, USA).

### 2.2. Mice

All procedures were performed in accordance with the Guide for the Care and Use of Laboratory Animals (Institute of Laboratory Animal Resources, 8th edition, 2011). Experimental protocols were approved by the Animal Care and Utilization Committee of National Yang-Ming University (Approval no. 1070314). Male apoE^−/−^ mice were obtained from the Jackson Laboratory (Bar Harbor, ME, USA). Mice were housed in barrier facilities under a controlled 12-h light/12-h dark cycle, with temperature maintained at 22 °C and humidity at 40–60%. Animals were fed a standard chow diet containing 4.5% fat (0.02% cholesterol) (Newco Distributors, Redwood, CA, USA). Four-month-old apoE^−/−^ mice were selected because they exhibit established hypercholesterolemia and early atherosclerotic lesions, allowing assessment of factors that accelerate disease progression rather than those that initiate lesions. Mice were orally administered KT-30 (5 mg/kg body weight) or vehicle (oil) daily for 4 weeks. At the end of treatment, mice were euthanized by CO_2_ inhalation. Body and organ weights were recorded. The livers, white adipose tissue (WAT), and brown adipose tissue (BAT) were collected for histological examination. Aortas and livers were homogenized to prepare lysates for Western blot analysis.

### 2.3. Histological Examination

Paraffin-embedded hearts, livers, WAT, and BAT were sectioned at a thickness of 8 µm for histological analysis. After deparaffinization, sections were stained with hematoxylin and eosin (H&E) and examined using a Motic TYPE 102M microscope (Motic Images, Xiamen, China). Quantification of atherosclerotic lesion area and assessment of cellular and non-cellular components were performed using Motic Images Plus 2.0 software.

### 2.4. Western Blot Analysis

Aortas and livers were lysed in an immunoprecipitation lysis buffer. Aliquots of lysates were separated by sodium dodecyl sulfate polyacrylamide gel electrophoresis (SDS-PAGE) and transblotted onto polyvinylidene difluoride (PVDF) membranes, blocked with 5% skim milk for 1 h at room temperature, then incubated with primary antibodies overnight and followed by the corresponding secondary antibodies for 3 h. Protein expression levels were detected using an enhanced chemiluminescence kit (PerkinElmer, Boston, MA, USA) and quantified in ImageJ 1.53 (NIH, Bethesda, MD, USA).

### 2.5. Measurement of Inflammatory Cytokines

Plasma levels of pro-inflammatory cytokines, including tumor necrosis factor-α (TNF-α), interleukin-1β (IL-1β), IL-6, and macrophage inflammatory protein-2 (MIP-2), were quantified using ELISA kits (R&D Systems, Minneapolis, MN, USA). Whole blood was collected, mixed with 10% (*v*/*v*) 3.2% sodium citrate, gently mixed, and allowed to stand for 10 min. Samples were then centrifuged at 1500× *g* for 10 min at 4 °C, and the resulting plasma supernatant was collected. All measurements were performed according to the manufacturer’s instructions.

### 2.6. MDA Measurement

Plasma and liver levels of the lipid peroxidation product malondialdehyde (MDA) were quantified using an MDA assay kit (700870; Cayman Chemical, Ann Arbor, MI, USA) according to the manufacturer’s instructions.

### 2.7. Serum Lipid Examination

Blood was collected via cardiac puncture. After clotting and centrifugation, serum was isolated, and levels of total cholesterol, high-density lipoprotein cholesterol (HDL-c), and triglycerides were measured using Spotchem EZ SP-4430 test strips (ARKRAY, Inc., Kyoto, Japan).

### 2.8. Measurements of Hepatic Lipids

Hepatic levels of total cholesterol, free cholesterol, cholesteryl esters (CE), triglycerides, fatty acids, and glycerol were determined using fluorometric assay kits (BioVision, Milpitas, CA, USA). Approximately 10 mg of liver tissue was used per assay, following the manufacturer’s protocols.

### 2.9. Liquid Chromatography–Tandem Mass Spectrometry (LC–MS/MS) Analysis

To explore the molecular mechanisms underlying KT-30-induced hepatic metabolic alterations, an exploratory discovery-based proteomic analysis was performed using LC–MS/MS. Because the primary objective was to identify candidate proteins and signaling pathways for subsequent biological validation, equal amounts of liver protein from each mouse in the same experimental group (*n* = 6 per group) were pooled into a single composite sample before analysis. Candidate proteins identified by the proteomic screening were subsequently validated by Western blot analyses using individual liver samples from each mouse.

The pooled protein samples were analyzed by Visual Protein Company (Taipei, Taiwan). Proteins were separated by 12.5% SDS-PAGE, stained with the VisPRO 5-min Protein Stain Kit, and stored at 4 °C. For in-gel digestion, each lane was excised into two slices and processed as described by Shevchenko et al. [30]. Gel pieces were washed, reduced with dithiothreitol (56 °C, 1 h), alkylated with iodoacetamide (room temperature, 45 min, in the dark), and digested overnight with Trypsin Gold. Peptides were extracted using 50% acetonitrile/1% formic acid, vacuum dried, and subjected to LC–MS/MS analysis.

Peptide separation was performed using an Ultimate 3000 nanoLC system (Thermo Fisher Scientific, Germering, Germany) equipped with a C18 PepMap column (75 μm × 25 cm, 2 μm, 100 Å) (Thermo Fisher Scientific, Sunnyvale, CA, USA) with a 90-min gradient (2–35% solvent B) at a flow rate of 300 nL/min and a column temperature of 35 °C. Mass spectrometric analysis was carried out on an Orbitrap Fusion Lumos mass spectrometer (Thermo Fisher Scientific, Waltham, MA, USA) operating in data-dependent acquisition mode. Full MS scans were acquired at a resolution of 120,000, followed by HCD MS/MS scans at a resolution of 15,000. Raw data were processed using Proteome Discoverer 2.5 with the MASCOT search engine against the UniProt Mouse database (17,131 sequences). Protein identification was performed using a precursor mass tolerance of 10 ppm and a fragment mass tolerance of 0.05 Da with a maximum of two missed tryptic cleavages. Carbamidomethylation was set as a fixed modification, whereas methionine oxidation, deamidation, and protein N-terminal acetylation were considered variable modifications. Peptide and protein identifications were accepted using a target-decoy strategy with a false discovery rate (FDR) of 0.05%, and only proteins with Mascot scores > 25 were included for subsequent analyses.

Because pooled samples, rather than individual biological replicates, were analyzed, the proteomic experiment was intended for qualitative discovery of candidate proteins rather than for statistical comparison of differential protein abundance between experimental groups. Candidate proteins were selected based on an absolute fold-change threshold of ≥1.5 and subsequently validated by Western blot analyses of individual liver samples. LC–MS/MS data were further analyzed using FunRich (v3.1.3) and Ingenuity Pathway Analysis (IPA, v76765844 M) to identify enriched biological pathways and molecular interaction networks. Canonical pathway enrichment was performed using the IPA algorithm. The reported *p* values represent pathway enrichment statistics generated by IPA and should not be interpreted as inferential statistical comparisons between biological replicates.

### 2.10. Statistical Analysis

Data are presented as mean ± SEM. Comparisons between two independent groups were performed using the nonparametric Mann–Whitney U test when the data did not meet the assumptions of parametric tests. Statistical analyses were conducted with SPSS version 20.0 (SPSS Inc., Chicago, IL, USA). A *p* value < 0.05 was considered statistically significant. For the exploratory proteomic analysis, no statistical comparisons of protein abundance were performed because pooled samples rather than biological replicates were analyzed. The reported FDR from Proteome Discoverer 2.5 was used solely to assess peptide and protein identification confidence and should not be interpreted as a statistical correction for differential protein expression.

## 3. Results

### 3.1. Effects of KT-30 on Body Weight, Organ Weight, Aortic Lesions, and Blood Lipids in apoE^−/−^ Mice

**Figure 1:**
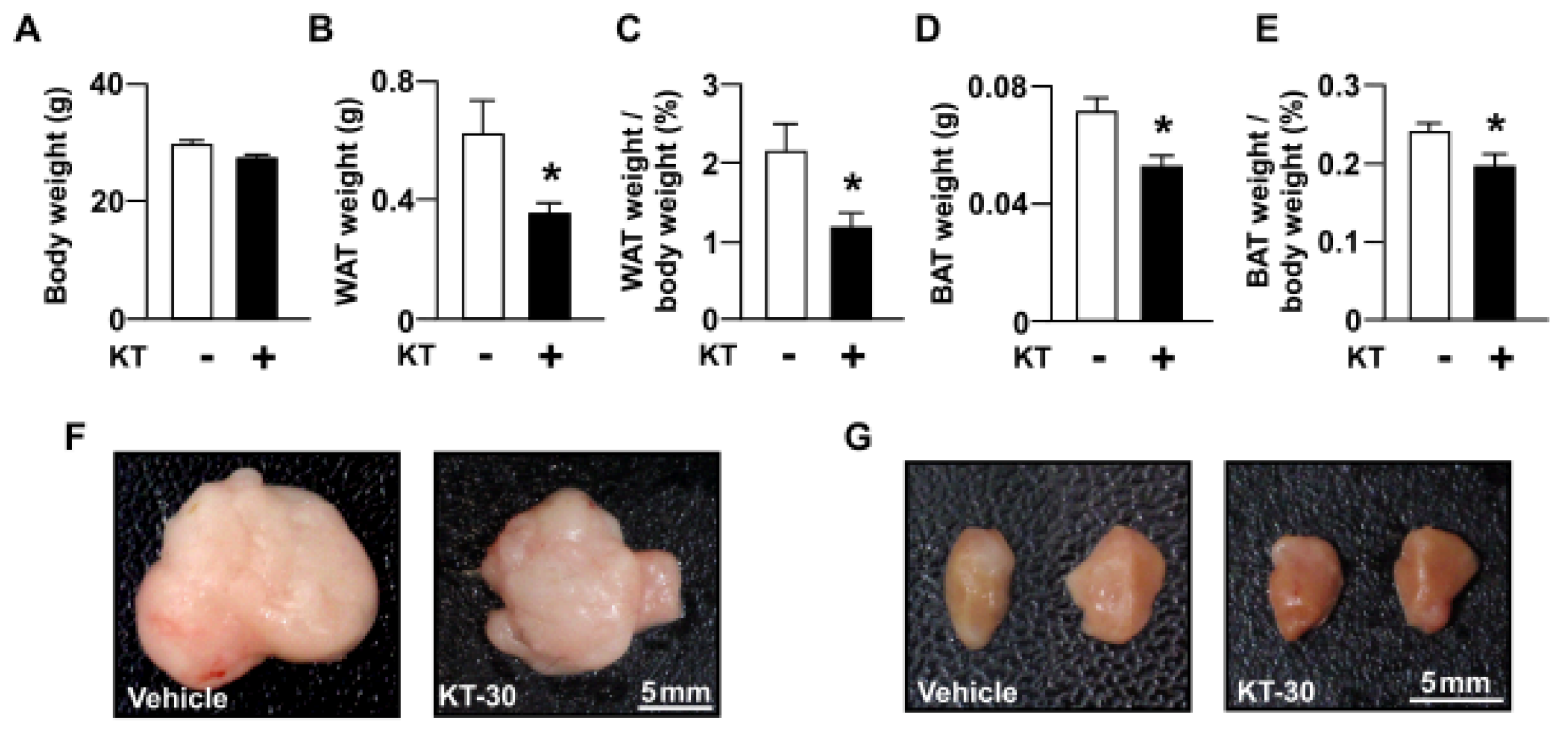
KT-30 reduces adipose tissue mass without affecting body weight in apoE^−/−^ mice. Male apoE^−/−^ mice at 16 weeks of age were orally administered KT-30 (5 mg/kg/day) or vehicle (oil) for 4 weeks. (**A**) Body weight. (**B**,**C**) The WAT weights and the ratios of WAT weight to body weight. (**D**,**E**) The BAT weights and the ratios of BAT weight to body weight. (**F**,**G**) The images of white adipose tissue (WAT) and brown adipose tissue (BAT). Data are presented as mean ± SEM (*n* = 6 per group). * *p* < 0.05 vs. vehicle.

**Figure 2:**
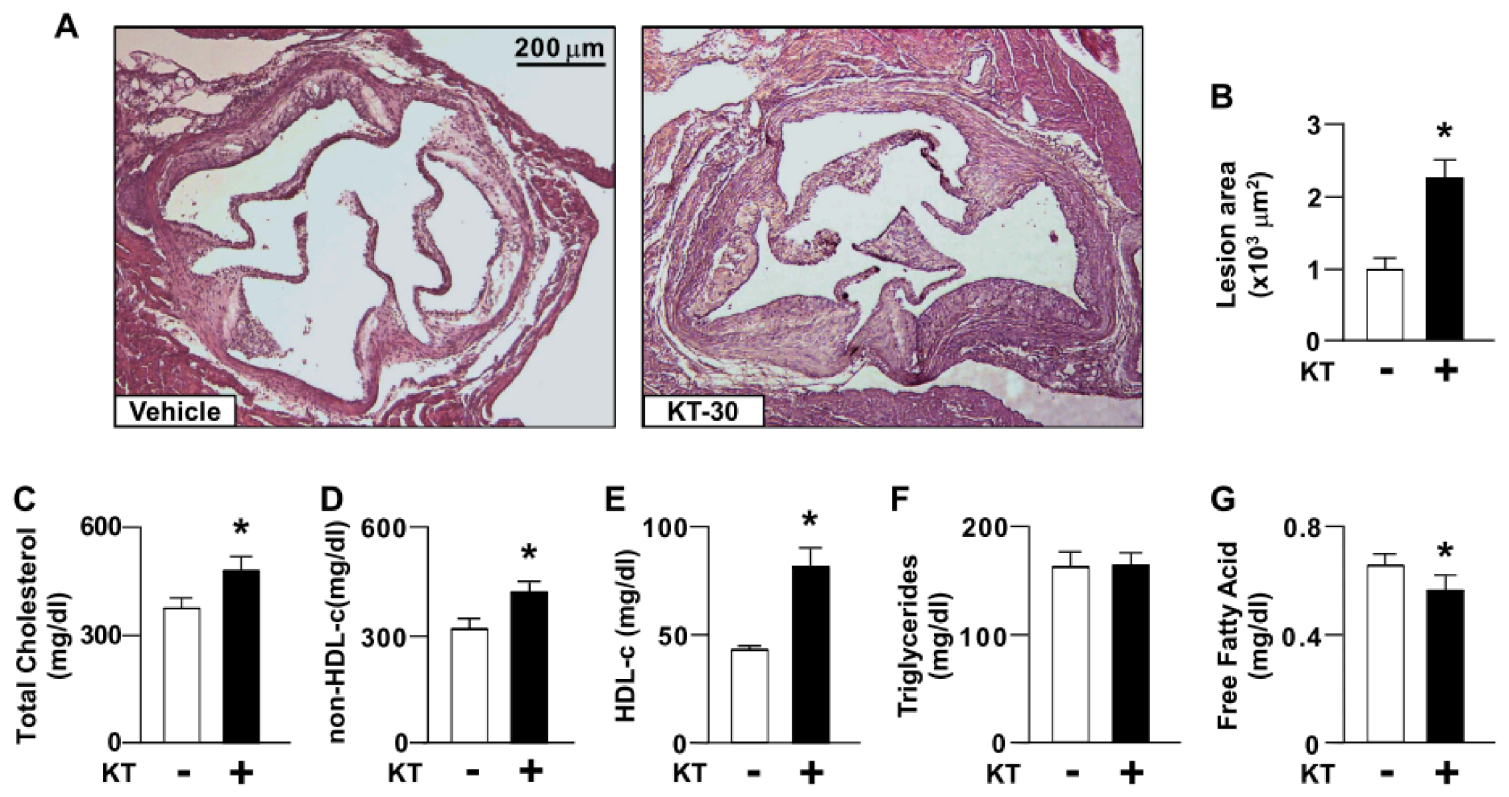
KT-30 accelerates atherosclerotic lesion formation and elevates plasma cholesterol levels. (**A**,**B**) Atherosclerotic lesions at the aortic roots were stained with H&E. Scale bar = 200 μM. (**C**–**E**) Plasma total cholesterol, non-HDL-cholesterol, and HDL-cholesterol levels were significantly increased in KT-30-treated mice. (**F**) Triglyceride levels were unchanged. (**G**) Plasma free fatty acid levels were reduced. Data are presented as mean ± SEM (*n* = 6 per group). * *p* < 0.05 vs. vehicle.

After 4 weeks of daily oral administration of KT-30 in 4-month-old apoE^−/−^ mice, there were no significant differences in body weight compared with the vehicle group (Figure 1A). We further analyzed WAT and BAT weights and observed a significant reduction in both depots in the KT-30 group compared with the vehicle group (Figure 1B–G). Moreover, KT-30 significantly increased aortic lesion formation (Figure 2A,B), indicating accelerated atherosclerosis in apoE^−/−^ mice. To investigate whether KT-30 affects lipid homeostasis, plasma lipids were measured. KT-30 treatment significantly increased total cholesterol, HDL-c, and non-HDL-c levels, whereas triglycerides remained unchanged (Figure 2C–F). Interestingly, free fatty acid levels were decreased in the KT-30 group (Figure 2G), suggesting that KT-30 disrupts cholesterol homeostasis. Together, these findings indicate that KT-30 alters systemic lipid distribution, increases total and non-HDL cholesterol levels, and promotes atherosclerosis progression.

### 3.2. Cholesterol Metabolism-Related Protein Expression Under KT-30 Administration

**Figure 3:**
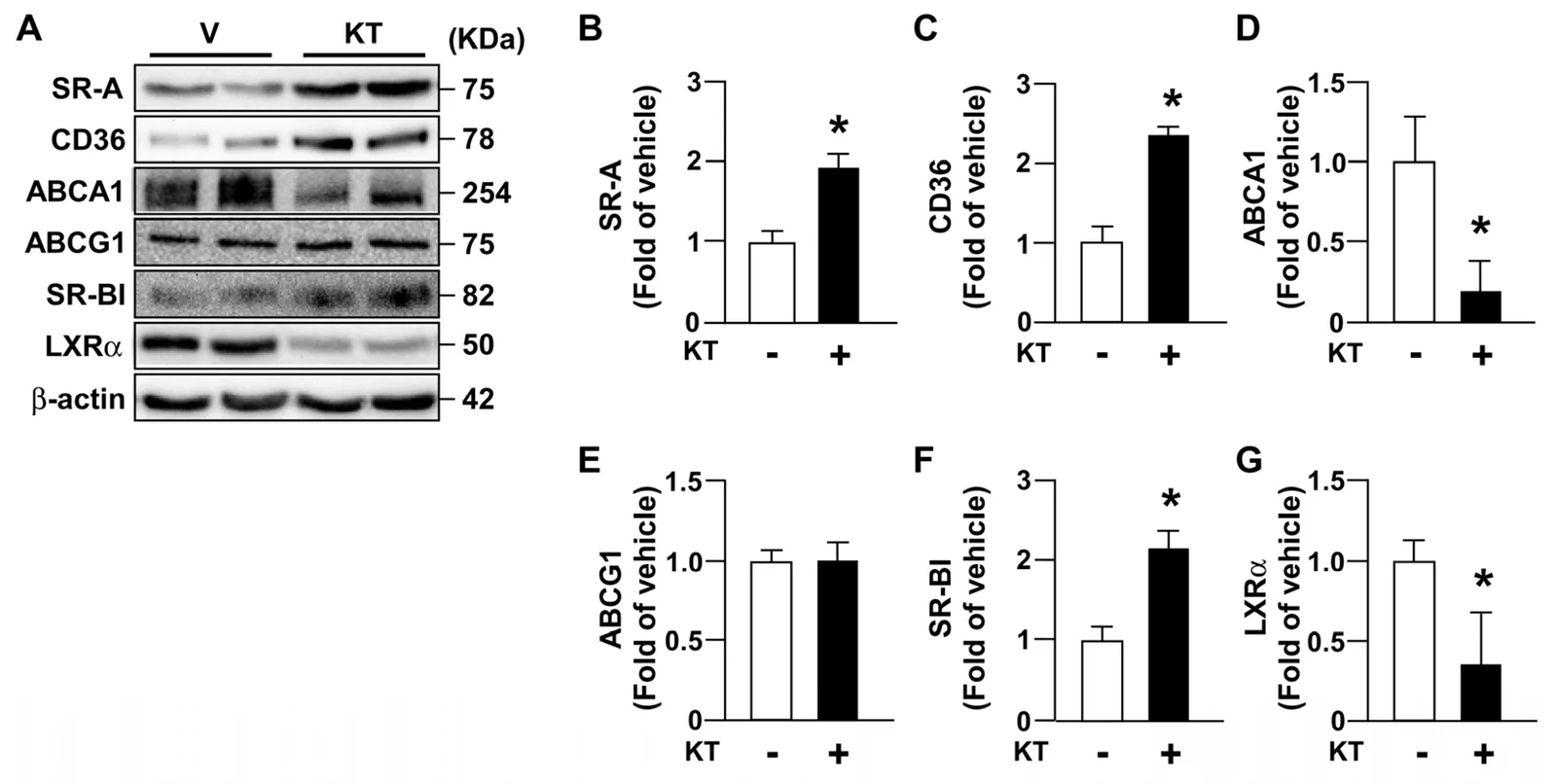
KT-30 alters aortic protein expression involved in cholesterol uptake and efflux. (**A**–**C**) Protein levels of scavenger receptors SR-A and CD36 were upregulated in the KT-30 group. (**D**–**G**) Expression of cholesterol efflux transporters ABCA1, as well as the regulator LXRα, was decreased, whereas SR-BI was increased. Representative Western blots and densitometric quantification are shown. Data are presented as mean ± SEM (*n* = 6 per group). * *p* < 0.05 vs. vehicle.

Cholesterol metabolism is a key factor for the development of atherosclerosis [31,32]. Macrophage receptors CD36 and SR-A mediate oxLDL uptake and foam cell formation, and promote atherosclerosis progression [33]. As shown in Figure 3A–C, KT-30 treatment increased aortic levels of CD36 and SR-A (Figure 3A–C). In contrast, cholesterol efflux in macrophages is mediated by SR-BI, ABCA1, and ABCG1 [34]. Our data showed that KT-30 significantly decreased ABCA1 protein levels and increased SR-BI protein expression without affecting ABCG1 protein levels (Figure 3A,D–F). Upstream, the transcription factor LXRα, a master regulator of cholesterol metabolism that controls ABCA1 gene expression [35,36], was decreased following KT-30 treatment (Figure 3A,G). These changes may reduce cholesterol efflux and enhance foam cell formation, exacerbating atherosclerosis.

### 3.3. KT-30 Increases Inflammatory Cytokines, MDA, and Inflammation-Related Protein Expression

**Figure 4:**
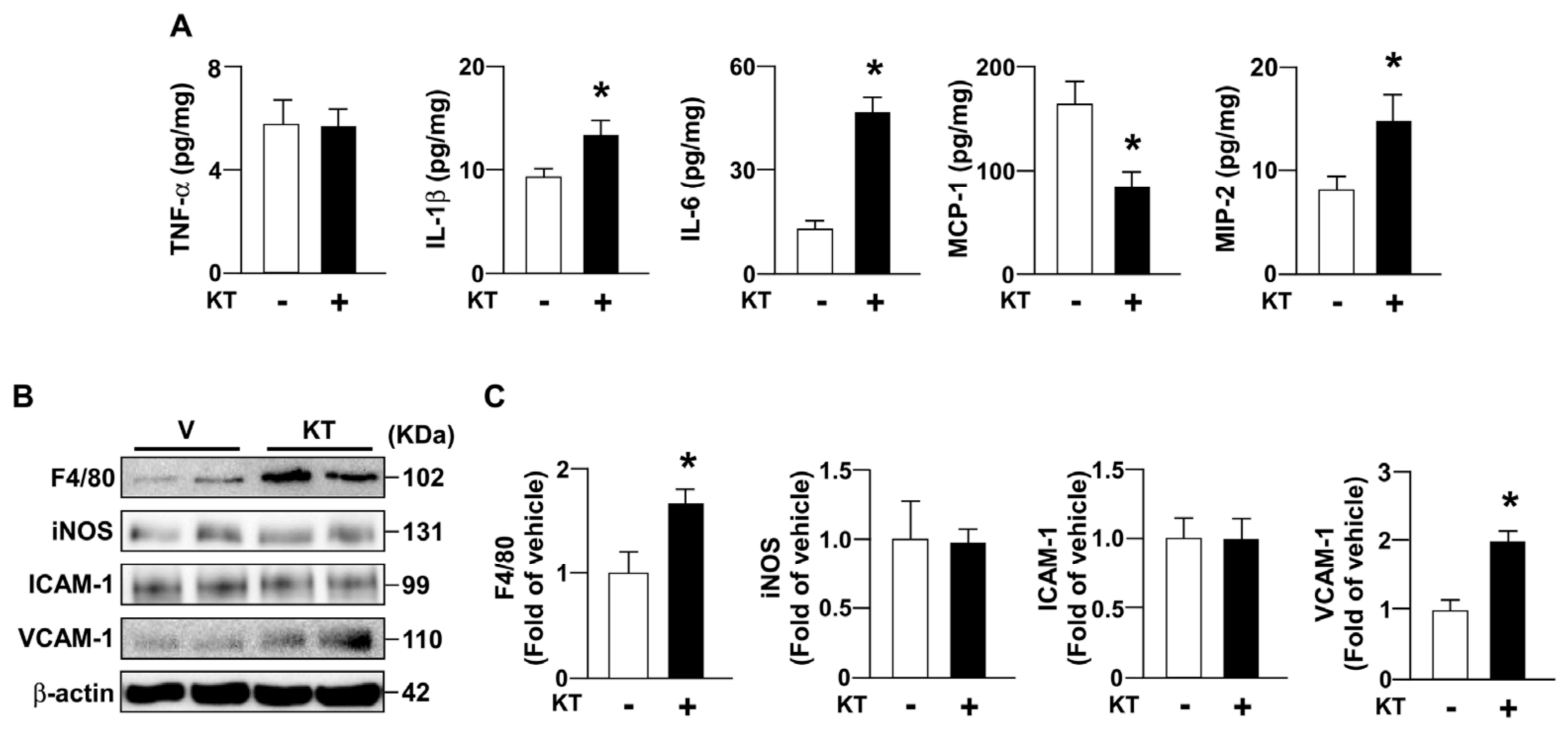
Levels of inflammation-related proteins and cytokines are altered after KT-30 administration. The expression levels of F4/80 (macrophage marker) and VCAM-1 were significantly elevated in the KT-30 group (**A**–**C**), whereas ICAM-1 and iNOS were unchanged. Levels of inflammatory cytokines IL-1β, IL-6, and MIP-2 were increased, whereas MCP-1 decreased, and TNF-α was unchanged. Data were represented as mean ± SEM from 6 mice. * *p* < 0.05 vs. vehicle group.

**Figure 5:**
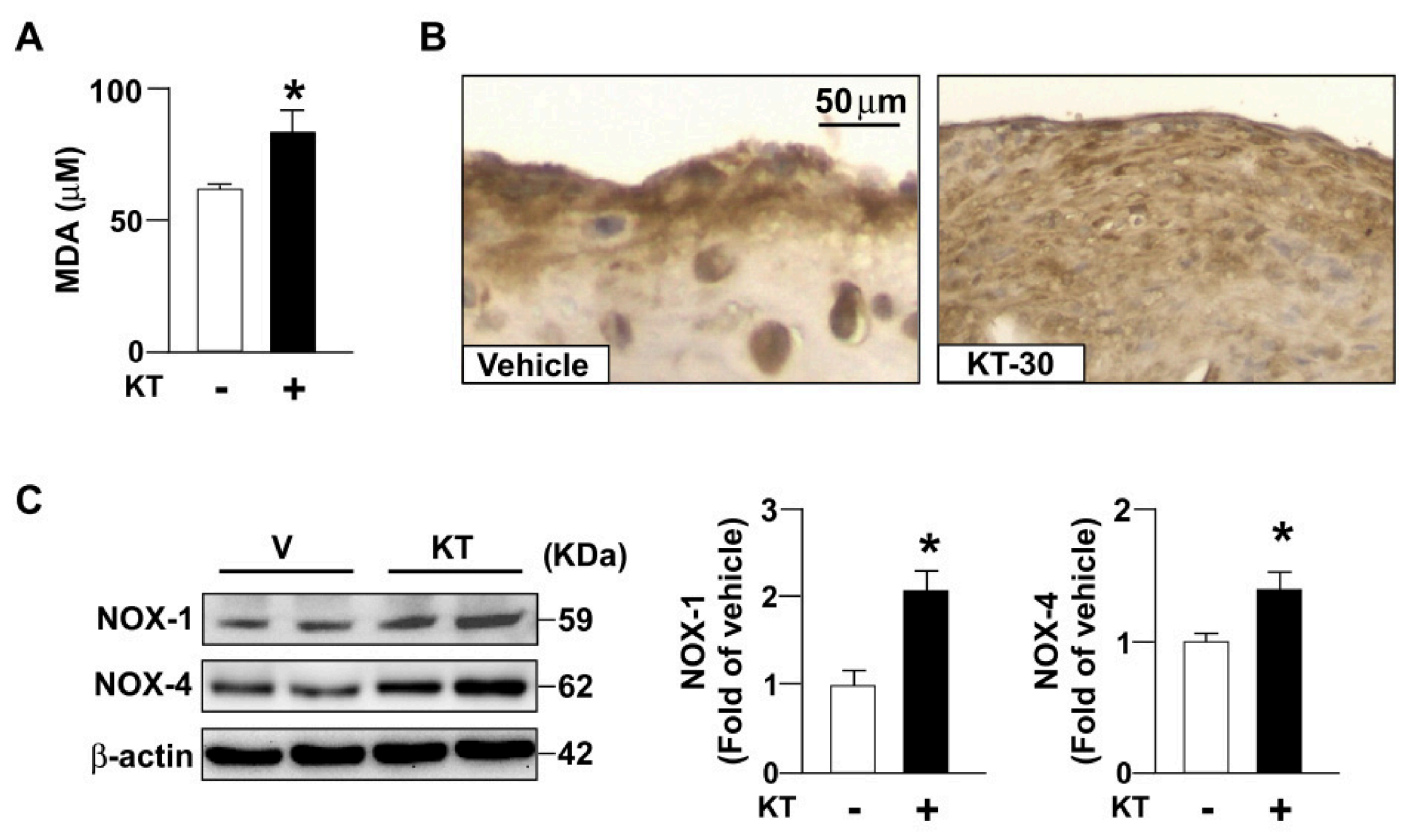
KT-30 promotes oxidative stress within atherosclerotic lesions. (**A**) Plasma levels of MDA. (**B**) Sections of atherosclerotic lesions were immunostained with normal rat IgG or an anti-4-HNE antibody. Cell nuclei were stained with hematoxylin. Scale bar = 50 μM. (**C**) Western blot analysis of NOX-1, NOX-4, and β-actin in atherosclerotic aortas. Data are presented as mean ± SEM (*n* = 6 per group). * *p* < 0.05 vs. vehicle.

The key inflammatory mediators, such as TNF-α, IL-1β, and MIP-2, play a crucial role in regulating vascular inflammation and the progression of atherosclerosis [37,38,39]. Compared with the vehicle group, 4-week administration of KT-30 elevated plasma levels of IL-1β, IL-6, and MIP-2 in apoE^−/−^ mice, whereas MCP-1 decreased and TNF-α remained unchanged (Figure 4A). Western blot analysis further revealed that KT-30 treatment elevated the levels of inflammation-related proteins F4/80 and VCAM-1 in the aortas of apoE^−/−^ mice, whereas iNOS and ICAM-1 levels were unchanged (Figure 4B,C). Similarly, plasma levels of the lipid peroxidation marker MDA and aortic levels of 4-HNE were increased in atherosclerotic lesions (Figure 5A,B). Furthermore, Western blot analysis indicated that KT-30 treatment raised the levels of oxidative stress-related proteins NOX-1 and NOX-4 in the aortas of apoE^−/−^ mice (Figure 5C). These findings suggest that KT-30 aggravates vascular inflammation and promotes atherosclerotic progression in apoE^−/−^ mice.

### 3.4. KT-30 Deregulates Hepatic Lipid Metabolism and Promotes Lipid Accumulation

**Figure 6:**
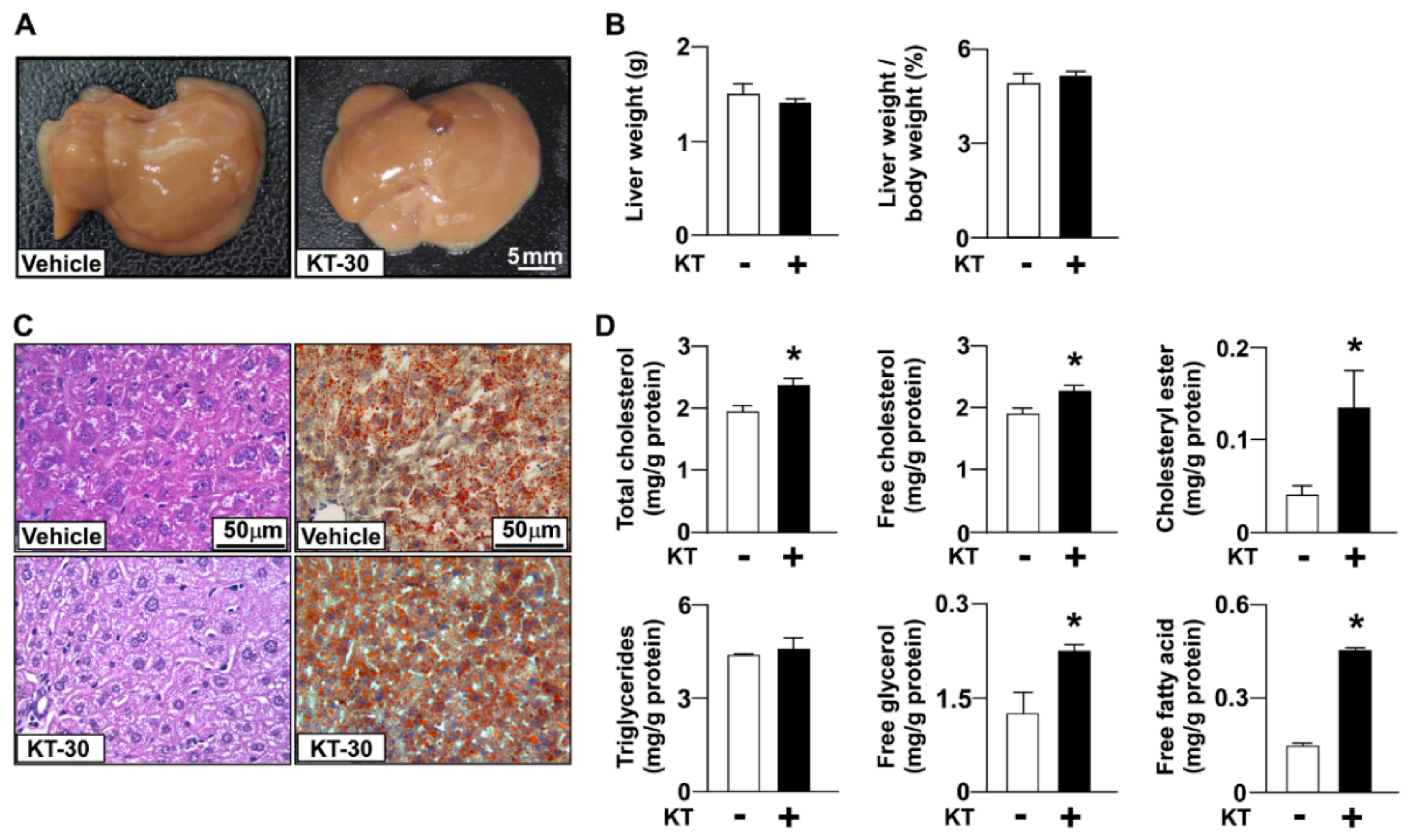
KT-30 induces hepatic lipid accumulation. (**A**,**B**) Liver weight and gross appearance were unaffected by KT-30 treatment. (**C**) H&E and Oil Red O staining revealed marked lipid droplet accumulation in the livers of KT-30-treated mice. (**D**) Hepatic levels of total cholesterol, free cholesterol, cholesteryl esters, free glycerol, and free fatty acids were significantly increased, whereas triglycerides were unchanged. Data are presented as mean ± SEM (*n* = 6 per group). * *p* < 0.05 vs. vehicle.

Administration of KT-30 did not affect liver weight in apoE^−/−^ mice (Figure 6A,B); however, histological analysis showed increased hepatic lipid accumulation. The histological sections with H&E-staining and Oil Red O staining revealed more lipid droplets in the liver of KT-30-treated apoE^−/−^ mice compared with the vehicle group (Figure 6C). Hepatic biochemical analysis indicated elevated levels of total cholesterol, free cholesterol, cholesteryl ester (CE), free glycerol, and free fatty acids, whereas triglycerides remained unchanged (Figure 6D). Because plasma lipid levels influence atherosclerosis progression [40,41] and are closely linked to hepatic lipid metabolism, these findings suggest that dysregulated hepatic lipid metabolism may contribute to KT-30-aggravated hyperlipidemia and atherosclerosis progression.

### 3.5. The Molecular Regulation of KT-30 in Disrupting Hepatic Lipid Homeostasis

**Figure 7:**
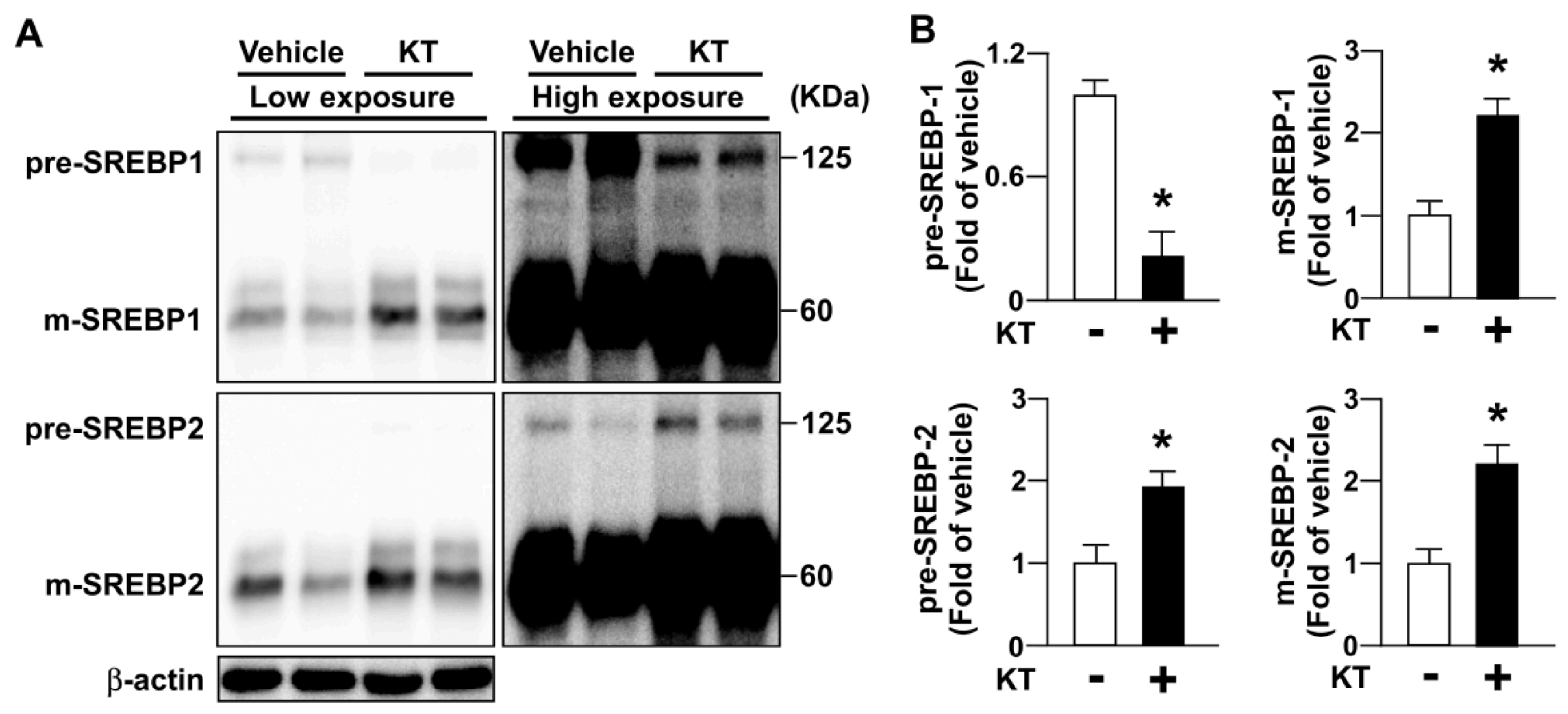
KT-30 upregulates hepatic regulators of de novo lipogenesis. (**A**,**B**) Protein levels of mature SREBP-1, precursor SREBP-2, and mature SREBP-2 were significantly increased in the livers of KT-30-treated mice, whereas precursor SREBP-1 was decreased. Representative Western blots and quantification are shown. Data are presented as mean ± SEM (*n* = 6 per group). * *p* < 0.05 vs. vehicle.

To further investigate the molecular mechanisms underlying the detrimental effects of KT-30 on hepatic lipid metabolism, we assessed the levels of proteins involved in de novo lipogenesis, lipoprotein influx/efflux, CE metabolism, biliary cholesterol secretion, fatty acid uptake, triglyceride synthesis, and mitochondrial β-oxidation in the livers of apoE^−/−^ mice. Western blot analysis showed that KT-30 treatment increased the expression of SREBP-1 and SREBP-2 (Figure 7), the key regulators of de novo lipogenesis [42,43], indicating that KT-30 enhances hepatic lipid synthesis.

**Figure 8:**
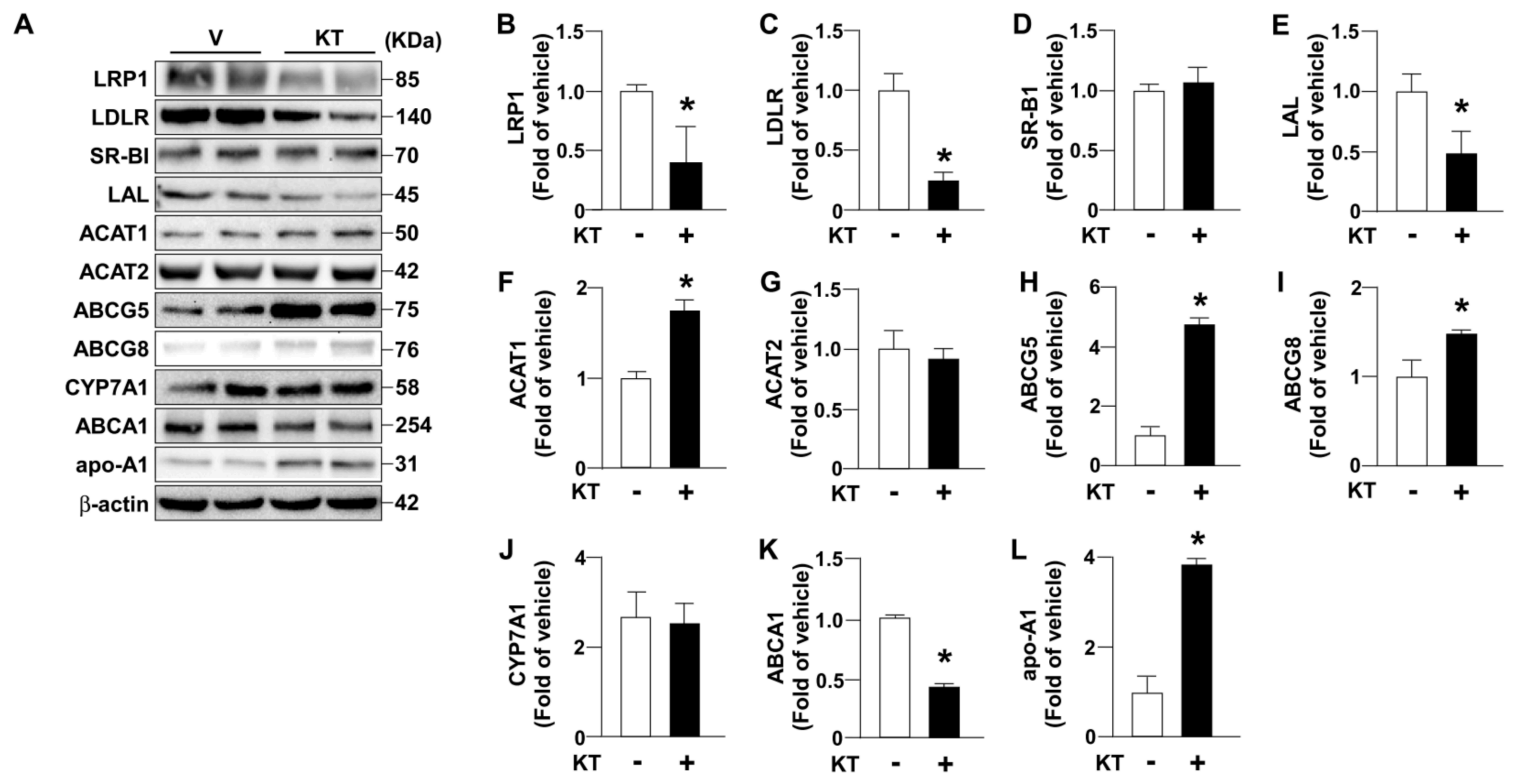
Effects of KT-30 on hepatic proteins involved in lipoprotein uptake, cholesterol ester metabolism, and biliary secretion. (**A**) Representative Western blot bands of target proteins and β-actin (loading control). (**B**–**L**) Quantitative analysis of relative protein expression levels for (**B**) LRP1, (**C**) LDLR, (**D**) SR-BI, (**E**) LAL, (**F**) ACAT1, (**G**) ACAT2, (**H**) ABCG5, (**I**) ABCG8, (**J**) CYP7A1, (**K**) ABCA1, and (**L**) apo-A1. Data are presented as mean ± SEM (*n* = 6 per group). * *p* < 0.05 vs. vehicle.

Additionally, treatment with KT-30 decreased LRP1, LDLR, LAL, and ABCA1, and increased ACAT1, ABCG5, ABCG8, and apo-AI, while SR-BI, ACAT2, and CYP7A1 were unchanged (Figure 8). LRP1, LDLR, and SR-BI are known to regulate lipoprotein uptake [44,45]; ACAT1, ACAT2, and LAL play key roles in controlling CE metabolism [46]; ABCG5, ABCG8, and CYP7A1 mediate biliary cholesterol secretion [47]; ABCA1 and apo-AI mediate HDL secretion [45,48]. These changes may explain the elevated blood lipids and hepatic cholesterol observed in KT-30-treated mice.

**Figure 9:**
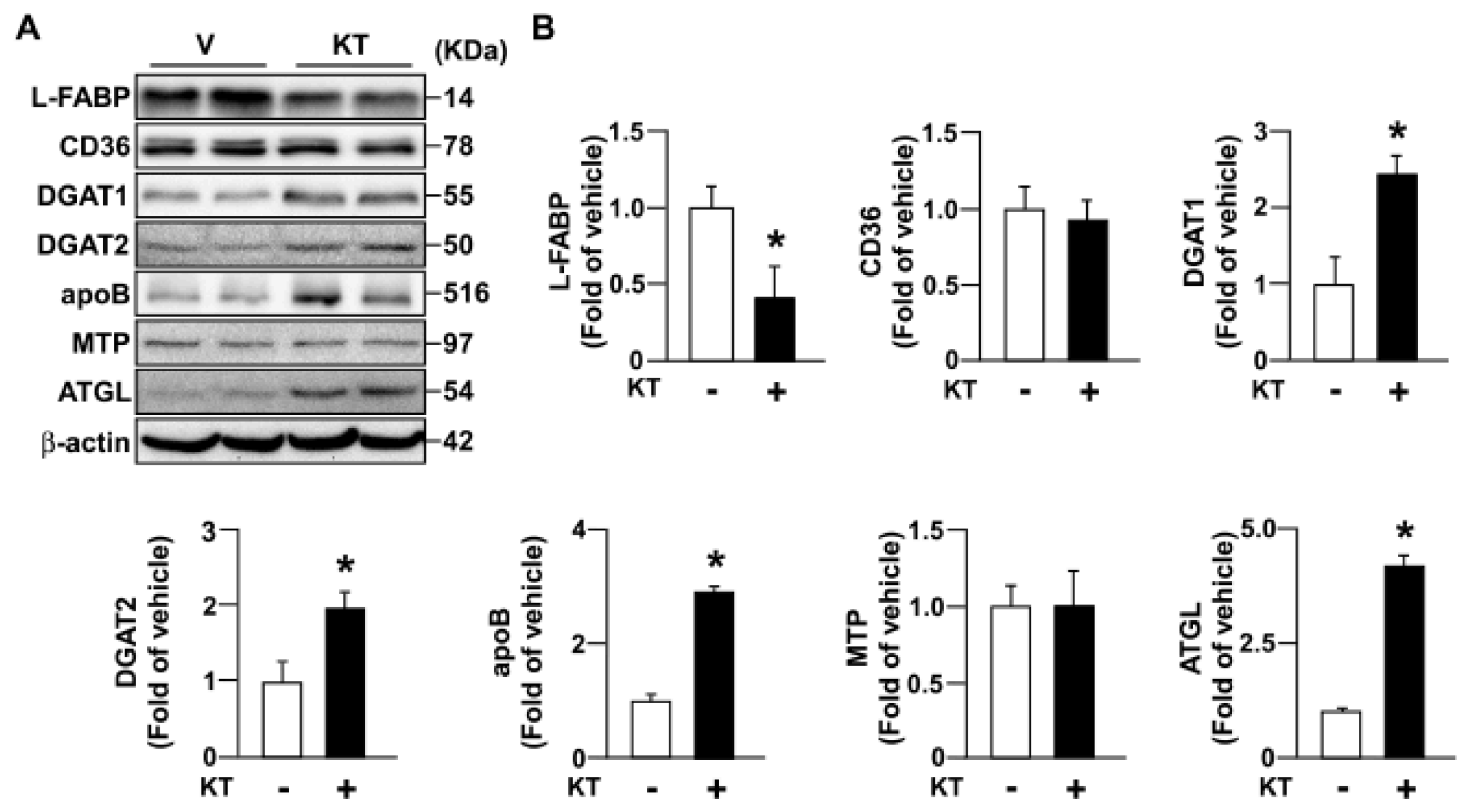
KT-30 alters hepatic triglyceride metabolism-related proteins. (**A**) Representative Western blot bands of target proteins and β-actin (loading control). (**B**) Quantitative analysis of relative protein expression levels for L-FABP, CD36, DGAT1, DGAT2, apoB, MTP, and ATGL. Data are presented as mean ± SEM (*n* = 6 per group). * *p* < 0.05 vs. vehicle.

**Figure 10:**
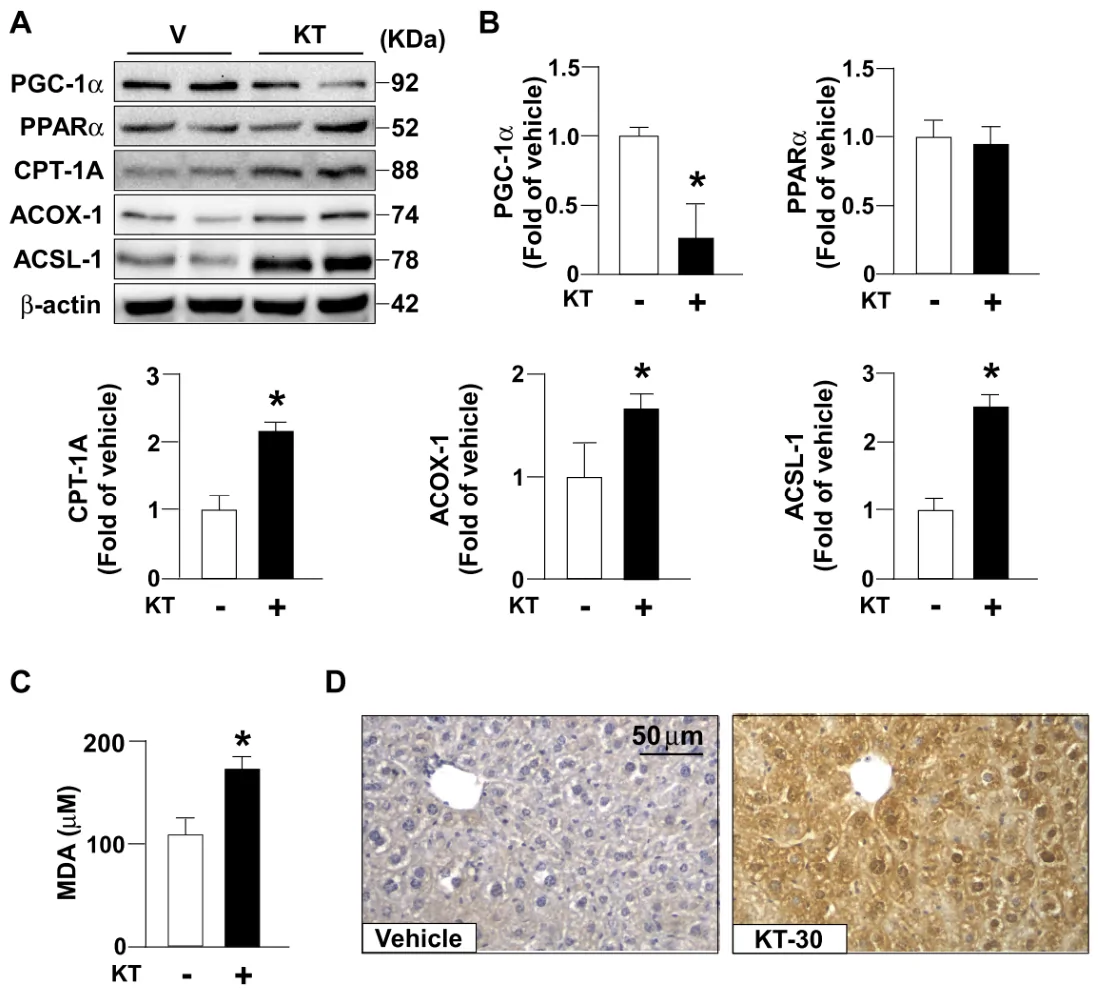
Effects of KT-30 on hepatic fatty acid β-oxidation-related proteins. (**A**,**B**) KT-30 decreased PGC-1α and increased CPT-1A, ACOX-1, and ACSL-1, whereas PPARα expression was unchanged. (**C**) Hepatic level of MDA. (**D**) The sections of the liver were immunostained with normal rat IgG or anti-4-HNE antibody. Cell nuclei were stained with hematoxylin. Scale bar = 200 μM. Data are presented as mean ± SEM (*n* = 6 per group). * *p* < 0.05 vs. vehicle.

In fatty acid uptake and triglyceride metabolism, L-FABP and CD36 regulate fatty acid transport [44], and KT-30 reduced L-FABP protein levels. Triglyceride synthesis and lipolysis, mediated by DGAT and ATGL [49,50], were increased. VLDL secretion, regulated by apoB [50,51], was also elevated (Figure 9). Moreover, mitochondrial lipid metabolism was disrupted, leading to increased oxidative stress. KT-30 treatment decreased PGC-1α, had no effect on PPARα, and increased CPT1A, ACOX-1, and ACSL1 levels, which are involved in mitochondrial function and fatty acid β-oxidation [45,52] (Figure 10A,B). Moreover, hepatic levels of the lipid peroxidation marker MDA and 4-HNE were increased (Figure 10C,D).

**Figure 11:**
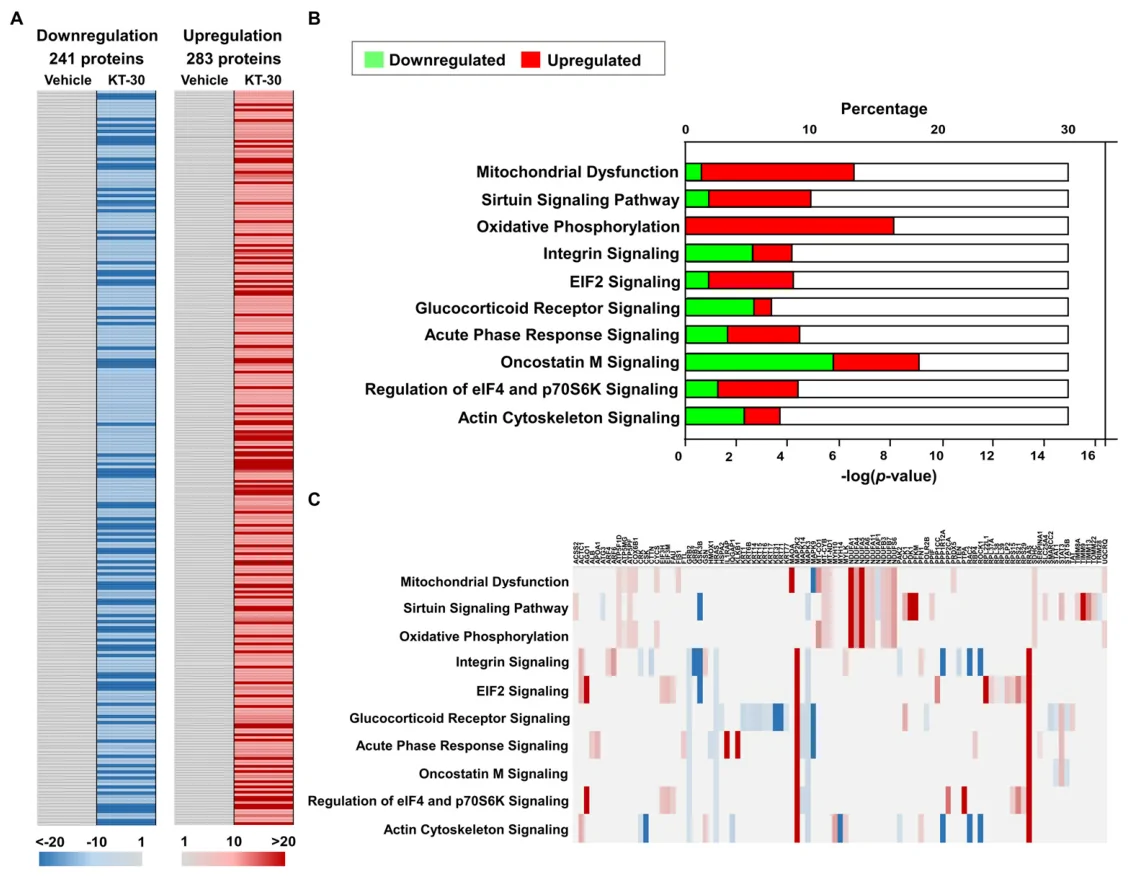
Distinct differentially expressed proteins and the top ten signaling pathways and related proteins affected by KT-30 in the liver of apoE^−/−^ mice. Male apoE^−/−^ mice at 16 weeks of age were orally administered KT-30 (5 mg/kg/day) or vehicle (oil) for 4 weeks. (**A**) Proteomic analysis using LC-MS/MS identified 283 upregulated and 241 downregulated proteins in the liver of KT-30-treated mice compared to the vehicle group. (**B**,**C**) The top ten canonical pathways were significantly enriched following KT-30 treatment, as determined by Ingenuity Pathway Analysis (IPA) using differentially expressed proteins identified by LC-MS/MS. Funrich V3.1.3 and IPA (76765844 M) to visualize molecular connections and canonical pathways altered by KT-30 treatment. Pathway enrichment and molecular network significance were defined as *p* < 0.05.

To gain mechanistic insight into the metabolic disturbances induced by KT-30, we performed an unbiased LC-MS/MS-based proteomic analysis using liver tissues. The liver was selected because it serves as the central organ regulating systemic lipid homeostasis, including cholesterol synthesis, fatty acid metabolism, lipoprotein production, and xenobiotic detoxification. In the present study, KT-30 exposure induced marked hepatic lipid accumulation, elevated hepatic cholesterol and fatty acid levels, and increased oxidative stress, suggesting that hepatic dysfunction may represent an upstream event contributing to the development of hyperlipidemia and atherosclerosis. Therefore, proteomic profiling was conducted to identify molecular pathways that may underlie KT-30-induced hepatic metabolic abnormalities. The proteomic analysis was performed using pooled samples as an exploratory screening approach; all major candidate proteins highlighted in the pathway analysis were subsequently validated using individual biological samples. As shown in Figure 11A, 283 proteins were upregulated, whereas 241 were downregulated. The ten most significantly altered signaling pathways were subsequently identified (Figure 11B). The differentially expressed proteins associated with each of these pathways, as determined by IPA, are presented in Figure 11C and Tables S1–S10.

## 4. Discussion

In this study, we investigated the atherogenic and metabolic impacts of the plant growth regulator KT-30 using apoE^−/−^ mice, a well-established model of atherosclerosis. Four weeks of KT-30 administration reduced WAT and BAT weights without affecting total body weight. Although this reduction in adipose tissue mass may superficially suggest a fat-loss effect, our findings demonstrate that KT-30 simultaneously elevates circulating cholesterol levels and accelerates atherosclerotic lesion development, indicating a detrimental rather than beneficial metabolic impact.

**Figure 12:**
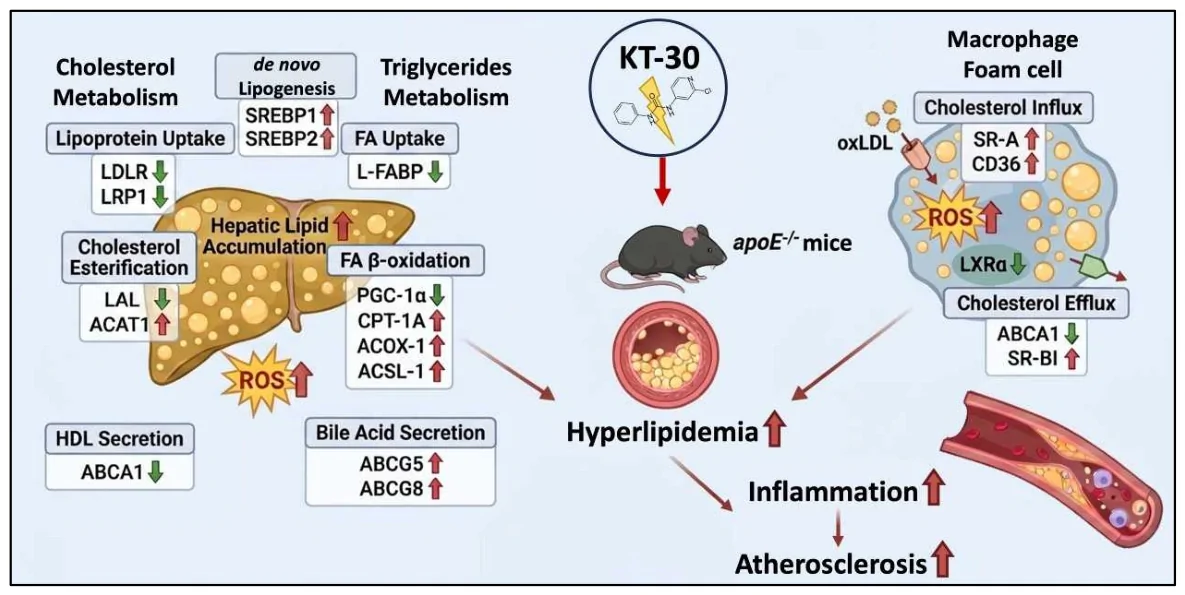
Schematic illustration of the proposed mechanisms by which KT-30 exposure accelerates atherosclerosis progression in apoE^−/−^ mice. Forchlorfenuron (KT-30), a widely used cytokinin-like plant growth regulator, simultaneously disrupts hepatic lipid homeostasis and promotes vascular oxidative stress, collectively driving hyperlipidemia, hepatic steatosis, and the progression of atherosclerosis. In the liver, KT-30 activates SREBP-1/2-mediated de novo lipogenesis, impairs lipoprotein uptake (LDLR ↓, LRP1 ↓) and HDL efflux (ABCA1 ↓), and enhances cholesterol esterification (ACAT1 ↑). Excessive mitochondrial β-oxidation generates disproportionate ROS, amplifying hepatic oxidative stress and lipid accumulation. In macrophages, KT-30 upregulates scavenger receptors (SR-A ↑, CD36 ↑) and suppresses LXRα-ABCA1-mediated cholesterol efflux, while ROS overproduction accelerates foam cell formation. These convergent hepatic and vascular oxidative insults drive hyperlipidemia, inflammation, and atherosclerosis progression in apoE^−/−^ mice. Red arrows indicate activation or up-regulation, while green arrows indicate inhibition or down-regulation.

KT-30 markedly upregulated the aortic expression of SR-A and CD36, two major macrophage scavenger receptors responsible for oxLDL uptake and foam cell formation [24]. This effect suggests enhanced lipid accumulation within macrophages, contributing to lesion progression. Beyond lipid uptake, cholesterol efflux also plays a pivotal role in maintaining vascular lipid homeostasis and preventing atherogenesis. The reduced expression of ABCA1, together with the downregulation of its upstream regulator LXRα [31,36], indicates impaired reverse cholesterol transport. Collectively, these findings suggest that KT-30 disrupts hepatic lipid homeostasis by downregulating lipoprotein uptake, fatty acid transport, CE hydrolysis, and HDL-mediated efflux, while upregulating CE and triglyceride synthesis. These key events may act in concert to disrupt hepatic lipid metabolism, thereby accelerating the progression of atherosclerosis (Figure 12). It should be noted that apoE^−/−^ mice inherently exhibit severe hypercholesterolemia and chronic low-grade vascular inflammation, rendering this model highly sensitive for evaluating factors that accelerate atherosclerosis progression. Consequently, the pro-atherogenic effects following KT-30 exposure may be amplified relative to those observed in metabolically healthy individuals. Therefore, caution should be exercised when extrapolating these findings directly to healthy human populations. Future studies using wild-type animals, diet-induced metabolic disease models, and long-term, environmentally relevant exposure paradigms will be important for determining the broader translational relevance of KT-30 exposure.

KT-30 also intensified systemic and vascular inflammation. Plasma levels of IL-1β, IL-6, and MIP-2 were elevated, consistent with their established roles in driving chronic vascular inflammation and plaque development [53,54]. Concurrently, KT-30 increased vascular expression of F4/80, VCAM-1, and NOX-1/4, all of which contribute to leukocyte recruitment, oxidative stress, and endothelial dysfunction [55]. VCAM-1 is a key mediator of monocyte adhesion and transmigration, serving as an early driver of plaque formation [54]. NOX-1 and NOX-4 promote vascular remodeling and oxidative stress via excessive ROS production [55]. The elevated plasma MDA levels, an end product of lipid peroxidation and a known risk factor for atherosclerosis [56], further support a role for KT-30 in exacerbating oxidative stress. Together, these inflammatory and oxidative alterations are consistent with a potential mechanistic contribution to the observed exacerbation of atherosclerosis in KT-30treated mice.

The KT-30induced reduction in adipose tissue mass appears paradoxical given the elevated plasma and hepatic lipid levels. Adipose tissue maintains systemic lipid homeostasis by regulating lipid storage and release through lipolysis [50,57]. The reduced WAT mass suggests enhanced lipolysis, potentially mobilizing stored lipids into circulation and contributing to the elevated plasma lipid burden. BAT, although relatively small in total mass, is metabolically active and vital for thermogenesis and triglyceride clearance [58,59,60,61]. The reduction in BAT weight suggests that KT-30 may suppress BAT physiological function, thereby impairing lipid clearance and contributing to dyslipidemia.

In the liver, KT-30 induced substantial lipid accumulation, as demonstrated by histological and biochemical analyses. The increased expression of SREBP-1 and SREBP-2 indicates activation of de novo lipogenesis, which accounts for a significant portion of hepatic lipid production [15]. This upregulation provides a mechanistic explanation for the elevated levels of hepatic cholesterol, cholesteryl esters, and fatty acids. Although proteins involved in lipoprotein and fatty acid uptake, including L-FABP and LRP1, were downregulated [44,48,52,62], KT-30 simultaneously increased the expression of DGAT1, DGAT2, and ACAT1, indicating enhanced triglyceride and cholesteryl ester synthesis. The elevated apoB level suggests increased VLDL secretion, thereby contributing to hyperlipidemia. This combination of increased lipid synthesis and lipid export aligns with the observed increase in blood lipid profile. Moreover, the elevated hepatic free fatty acid levels following KT-30 treatment may stimulate β-oxidation, as indicated by the upregulation of CPT-1A, ACOX-1, and ACSL-1. While β-oxidation provides energy, excessive activation generates ROS [55,56], potentially contributing to oxidative stress, inflammation, and liver injury. Thus, KT-30 disrupts multiple components of hepatic lipid metabolism, culminating in lipid accumulation and oxidative stress. Overall, the aortic, plasma, adipose, and hepatic findings converge to demonstrate that KT-30 profoundly disrupts systemic lipid homeostasis and accelerates atherogenesis.

These apparently paradoxical findings likely reflect a compensatory but insufficient metabolic response rather than a simple lipogenic phenotype. The reduction in plasma FFA alongside increased hepatic FFA suggests enhanced hepatic FFA uptake/trapping that outpaces peripheral release. Similarly, the concurrent upregulation of ATGL (lipolysis) and CPT1A/ACOX1 (β-oxidation) indicates that the liver is actively attempting to mobilize and oxidize the excess lipid burden; however, this compensatory oxidative flux is evidently insufficient to offset the markedly increased rate of SREBP-driven de novo lipogenesis and lipoprotein/cholesterol uptake, resulting in net lipid accumulation. This pattern is consistent with an “adaptive overload” state described in other models of hepatic steatosis, in which upregulated β-oxidation machinery fails to prevent triglyceride/FFA accumulation because substrate delivery exceeds oxidative capacity.

Notably, IPA canonical pathway analysis identified the “Mitochondrial Dysfunction” pathway as one of the most significantly enriched pathways and oxidative phosphorylation (OXPHOS) as the most significantly enriched IPA canonical pathways (Tables S1–S3), based on coordinated changes in the abundance of pathway-associated proteins; as no direct functional mitochondrial assays (e.g., oxygen consumption, ATP production, or membrane potential) were performed, these findings indicate alterations in mitochondrial pathway-related protein expression rather than confirmed functional mitochondrial impairment. Accordingly, the present findings should be interpreted as evidence of mitochondrial pathway remodeling and altered mitochondrial homeostasis rather than definitive proof of mitochondrial dysfunction. Future studies incorporating direct functional mitochondrial assays will be required to determine whether these proteomic alterations translate into impaired mitochondrial bioenergetics and respiratory capacity.

KT-30 coordinately upregulated all five electron transport chain (ETC) complexes (multiple NADH dehydrogenase, cytochrome b/c, COX, succinate dehydrogenase, and ATP synthase subunits, etc.), likely reflecting a compensatory response to bioenergetic insufficiency rather than augmented capacity [63,64]. Paradoxically, elevated ETC flux in the context of excess fatty acid influx generates disproportionate superoxide at Complexes I and III [61], worsening lipid peroxidation and mitochondrial DNA damage. Elevation of pro-apoptotic cytochrome c (>2-fold) and the mitochondrial fission protein FIS1 suggests fragmentation toward apoptotic susceptibility [64], while upregulation of the antioxidant peroxiredoxin-5 reflects an inadequate compensatory scavenging response [65]. Downregulation of ATG3 (Table S2) impairs mitophagy, allowing dysfunctional, ROS-generating mitochondria to accumulate, thereby perpetuating oxidative injury [65,66]. The sirtuin signaling pathway (Table S2) was enriched with upregulated mitochondrial import translocases (Tim8A, Tim9, Tim13) and ANT1, alongside PEPCK and PFK-M, indicating metabolic reprogramming toward gluconeogenesis and glycolysis consistent with an energy-deficient state [67]. STAT3 upregulation (>3-fold) via the JAK–STAT3 axis promotes hepatic lipogenesis and suppresses fatty acid oxidation [68], while downregulation of GSK-3β, ERK1, and ATG3 reflects impaired insulin signaling and autophagic quality control. Together, these changes disrupt mitochondrial homeostasis and amplify lipid accumulation by simultaneously activating lipogenic transcription and impairing mitophagy.

Integrin signaling (Table S4) showed potent upregulation of MEK2 and R-Ras (>10-fold), along with cytoskeletal proteins gelsolin, profilin-1, and MLCK, consistent with increased contractility and a pro-fibrotic phenotype [67,69]. PTEN downregulation (−3.24-fold) disinhibits mTORC1/SREBP-1c-driven lipogenesis, and concurrent loss of PAK2, GRB2, and FAK-dependent signaling impairs hepatocyte polarity and biliary cholesterol secretion [47]. In the EIF2 signaling pathway (Table S5), broad upregulation of ribosomal subunits (40S and 60S) and eIF3M/H reflects compensatory translational capacity under oxidative stress, while AGO1 induction (>10-fold) reprograms miRNA-mediated silencing of hepatic lipid genes (e.g., miR-122, miR-33) [41]. PP1-γ upregulation resolves the integrated stress response, enabling bulk translation to resume despite ongoing ROS production [67,70].

Glucocorticoid receptor signaling (Table S6) showed upregulation of PEPCK (4.29-fold) and TAT (2.24-fold), consistent with glucocorticoid-driven gluconeogenesis, alongside STAT3 elevation and STAT1/STAT5B suppression [69]. Downregulation of multiple keratins and SMARCC2 suggests epigenetic dysregulation and intermediate-filament collapse, impairing hepatocyte resilience. In the acute phase response (Table S7), upregulation of IL-1RAcP (>10-fold) and plasma kallikrein (>10-fold) amplifies IL-1β-driven inflammation and promotes a pro-thrombotic state [54]. Despite ApoA-I elevation (3.85-fold), ABCA1 impairment [43] indicates dysfunctional HDL production. Critically, HO-1 downregulation (−2.54-fold) removes a key cytoprotective brake, directly linking hepatic APR changes to aortic NOX-1/4 upregulation and elevated plasma MDA [55].

Oncostatin M signaling (Table S8) revealed a STAT3-dominant/STAT1-STAT5B-deficient hepatic environment that amplifies lipogenesis and impairs GH-dependent fatty acid oxidation [68]. eIF4/p70S6K signaling (Table S9) showed marked PP2A upregulation (>10-fold) suppressing mTORC1 effectors [67], co-existing with elevated AGO1 and ribosomal S21, reflecting context-dependent translational reprogramming under metabolic stress [70]. Finally, actin cytoskeleton signaling (Table S10) demonstrated upregulation of myosin-10 (5.08-fold), MLCK, gelsolin, and profilin-1 alongside profound downregulation of IQGAP1 (−3.89-fold), PAK2, cortactin, and Rac2 [69]. These changes promote cytoskeletal stiffening and junctional disruption, impairing bile canalicular secretion, lipoprotein transcytosis, and hepatocyte barrier integrity, thereby increasing endotoxin permeability and amplifying inflammatory signaling.

Collectively, the ten proteomic pathways (Tables S1–S10) converge on oxidative stress as a central unifying theme. The pathway-level protein signature is consistent with mitochondrial electron leak (Tables S1–S3) that may be perpetuated by impaired mitophagy (ATG3 loss, Table S2), loss of HO-1 cytoprotection (Table S7), and STAT3-driven lipogenic amplification (Tables S2, S6–S9) [60,61,65,66]. This proposed ROS burden would be expected to engage the integrated stress response (Table S5), the glucocorticoid and acute-phase cascades (Tables S6 and S7), and potentially disrupt cytoskeletal integrity (Tables S4 and S10), thereby potentially increasing hepatocyte permeability and further activating NF-κB/STAT3 inflammatory loops [67,69,70]. We propose that a feed-forward cycle links mitochondrial oxidative stress to SREBP-dependent lipogenesis [15,61], producing a coherent molecular framework that parallels the vascular oxidative burden (elevated plasma MDA, aortic 4-HNE, and upregulation of NOX-1/4) observed alongside hepatic steatosis and accelerated atherosclerosis progression; as no antioxidant, NOX-inhibition, or genetic suppression intervention was performed, this framework should be regarded as a hypothesis-generating mechanistic model rather than a causally established pathway.

A major objective of the proteomic analysis was to determine how KT-30 disrupts hepatic metabolic homeostasis, thereby promoting a pro-atherogenic environment. The liver is the primary organ responsible for coordinating systemic cholesterol and lipid metabolism. Consequently, hepatic dysfunction can profoundly influence circulating lipid profiles and accelerate the development of atherosclerosis. In the current study, KT-30 induced hepatic lipid accumulation and altered the expression of proteins involved in lipid metabolism, oxidative stress responses, and xenobiotic processing. These findings suggest that the liver is a critical target organ of KT-30 toxicity and may function as an upstream regulator of the metabolic abnormalities observed in KT-30-treated apoE^−/−^ mice. Importantly, atherosclerosis is increasingly recognized as a systemic metabolic disease rather than solely a vascular disorder [10,11,12]. Disturbances in hepatic lipid handling can increase circulating atherogenic lipoproteins, promote chronic inflammation, and enhance oxidative stress, all of which contribute to plaque progression [12,71,72,73]. Therefore, our proteomic analysis was designed to identify hepatic mechanisms linking KT-30 exposure to dyslipidemia and accelerated atherosclerosis. While proteomic analyses of aortic tissue or plasma may provide additional information regarding local vascular responses and circulating factors, the present study focused on the liver because it exhibited the most prominent metabolic alterations and represents a biologically relevant upstream driver of atherosclerotic disease progression.

Although the present study focused on characterizing pro-oxidant markers rather than directly measuring antioxidant enzyme activity, our proteomic data provide indirect insight into the antioxidant status of KT-30-exposed tissues. The observed dysregulation of sirtuin signaling is particularly notable, as sirtuins, especially SIRT1 and SIRT3, are key regulators of mitochondrial redox homeostasis, promoting the expression of antioxidant enzymes such as SOD2 and catalase through FOXO3a and PGC-1α-dependent pathways [74,75]. Impairment of this axis, together with the upregulation of NOX-1/4, suggests that KT-30 exposure not only increases ROS production but may also compromise endogenous antioxidant defense capacity, creating a pro-oxidative imbalance that facilitates lipid peroxidation and atherosclerotic progression. These findings raise the possibility that antioxidant-based strategies, such as NOX inhibitors, mitochondria-targeted antioxidants, or sirtuin activators, could mitigate KT-30-induced vascular and hepatic oxidative injury, a hypothesis that warrants direct testing, including SOD/GPx activity assays and Nrf2 pathway analysis, in future studies.

An important finding of the present study is the close association between KT-30-induced hepatic lipid accumulation and accelerated atherosclerosis. The liver is the primary organ responsible for maintaining systemic lipid homeostasis by regulating cholesterol synthesis, fatty acid metabolism, lipoprotein production, and bile acid metabolism. Consequently, hepatic metabolic dysfunction can profoundly influence circulating lipid levels and cardiovascular risk. Excessive hepatic lipid accumulation promotes the overproduction of atherogenic lipoproteins, impairs cholesterol disposal pathways, and enhances oxidative stress and inflammatory signaling. These alterations contribute to dyslipidemia and increase the delivery of cholesterol-rich lipoproteins to the arterial wall, thereby facilitating foam cell formation and atherosclerotic plaque progression. In the present study, KT-30 exposure induced hepatic lipid accumulation, increased hepatic cholesterol and fatty acid contents, elevated circulating cholesterol levels, and enhanced atherosclerotic lesion formation. These findings suggest that hepatic metabolic disturbances may represent an upstream mechanism contributing to KT-30-induced atherogenesis. Consistent with this hypothesis, LC-MS/MS analysis identified significant alterations in proteins involved in lipid metabolism, oxidative stress responses, and xenobiotic processing, further supporting the notion that disruption of hepatic metabolic homeostasis plays a central role in the pro-atherogenic effects of KT-30.

Several limitations of the present study should be acknowledged. First, although atherosclerotic lesion burden was quantified by cross-sectional analysis of the aortic root, which is a well-established and widely accepted method for assessing atherosclerosis in apoE^−/−^ mice, en face analysis of the entire aorta was not performed. Therefore, regional lesion distribution throughout the arterial tree could not be evaluated. This experimental design was adopted because the arterial tissues were preserved for subsequent protein extraction and mechanistic studies, thereby enabling assessment of both histological and molecular endpoints from the same cohort of animals. This approach was consistent with the principles of the 3Rs, particularly the Reduction principle, by maximizing scientific output while minimizing animal use. Nevertheless, future studies incorporating both aortic root and en face analyses would provide a more comprehensive assessment of atherosclerotic burden and lesion distribution. Second, although mRNA expression analyses were not performed, the present study focused primarily on protein abundance, as the biological processes investigated are ultimately mediated by protein function. Future studies integrating transcriptomic analyses may provide additional mechanistic insight into the regulation of KT-30-induced molecular alterations.

Third, this study was performed in a single genetically susceptible apoE^−/−^ mouse model, which was selected to investigate factors accelerating atherosclerosis progression and may not fully represent the complexity of human cardiovascular disease. Fourth, the 4-week KT-30 treatment used in this study represents a subacute, rather than a chronic, dietary exposure paradigm. Although this exposure duration was sufficient to demonstrate significant biological effects, longer-term studies using environmentally relevant exposure levels would better mimic chronic human dietary exposure and allow assessment of the cumulative cardiovascular consequences of prolonged KT-30 exposure. Fifth, although the administered dose was selected based on previous toxicological studies, its relationship to typical human dietary exposure remains uncertain.

Sixth, only male apoE^−/−^ mice were included in the present study. Although this experimental design facilitated mechanistic investigation in an established model of atherosclerosis, sex is increasingly recognized as an important biological variable influencing lipid metabolism, oxidative stress, inflammatory responses, and susceptibility to atherosclerosis. Consequently, the present findings should not be assumed to be directly applicable to females. Future studies including both male and female animals will be required to determine whether KT-30 exerts sex-dependent cardiovascular and metabolic effects. Seventh, the LC-MS/MS proteomic analysis was performed using pooled liver protein samples from each experimental group. Although this strategy facilitated the exploratory identification of candidate proteins and pathways associated with KT-30 exposure, it precluded assessing biological variability and statistically evaluating differential protein expression among individual animals. Therefore, the proteomic results should be interpreted as hypothesis-generating rather than definitive evidence. Importantly, the principal candidate proteins identified by the proteomic screening were subsequently validated by Western blot analyses using individual liver samples, thereby strengthening the reliability of the major mechanistic conclusions. Future studies employing quantitative proteomic analyses with biological replicates will be valuable for further validating and extending these findings. Finally, no measurements of internal KT-30 concentrations or exposure biomarkers were performed, and no human biomonitoring or epidemiological evidence is currently available. Therefore, the present findings should be interpreted as providing experimental evidence and biological plausibility for KT-30-induced pro-atherogenic effects rather than establishing direct human cardiovascular risk. Future studies incorporating environmentally relevant exposure levels, chronic exposure paradigms, human biomonitoring, epidemiological investigations, and both sexes will be important for determining the translational significance of these findings.

One important limitation of this study concerns the dose used and its translational relevance to human exposure. The dose of KT-30 selected in the present study (5 mg/kg/day) was determined from preliminary in vitro cell-based experiments that identified this concentration as biologically effective in disrupting lipid metabolism. Although no prior studies have systematically examined the effects of KT-30 on human health, this compound is widely used in agriculture to enhance fruit size and commercial value, raising legitimate concerns about chronic dietary exposure in the general population. To provide translational context, the human equivalent dose (HED) was estimated using the FDA-recommended body surface area (BSA)-based conversion formula, HED = animal dose × (animal Km/human Km), where Km values for mice and humans are 3 and 37, respectively. Applying this formula, the HED corresponding to the mouse dose of 5 mg/kg/day is approximately 0.4 mg/kg/day, equivalent to roughly 24 mg/day for a 60 kg adult. We acknowledge that this estimated human equivalent dose likely exceeds typical dietary KT-30 exposure from fruit consumption under normal conditions, which represents a limitation of the current study. Nevertheless, the primary objective of this investigation was to establish a proof-of-concept framework demonstrating that KT-30 possesses the biological capacity to disrupt lipid homeostasis and accelerate atherosclerosis progression through well-defined molecular mechanisms. This approach is consistent with initial mechanistic studies of other agrochemicals and food-associated compounds, in which supratherapeutic doses are employed to reveal biological pathways before lower-dose, environmentally relevant investigations are conducted. Furthermore, given that individual dietary habits, cumulative exposure from multiple fruit sources, and potential long-term low-dose effects remain poorly characterized, the actual risk posed by KT-30 to cardiovascular health cannot be fully ruled out at this stage. Future studies incorporating environmentally relevant concentrations and dose–response experimental designs are warranted to more precisely define the threshold at which KT-30 exposure may pose a meaningful cardiovascular risk in humans.

To further contextualize the translational relevance of the administered dose, we compared our estimated HED with established regulatory exposure thresholds. The European Food Safety Authority (EFSA) has established an acceptable daily intake (ADI) for forchlorfenuron of 0.05 mg/kg bw/day, based on a 2-year mouse study, together with an acute reference dose (ARfD) of 1 mg/kg bw and an acceptable operator exposure level (AOEL) of 0.25 mg/kg bw/day [76]. The HED for our administered dose (approximately 0.4 mg/kg/day) therefore exceeds the EFSA ADI by approximately 8-fold, while remaining below the ARfD. Notably, EFSA’s dietary exposure modeling, based on established maximum residue limits for kiwifruit and grapes (0.01–0.05 mg/kg), estimates that real-world chronic dietary intake of forchlorfenuron accounts for less than 1% of the ADI [76], indicating that the dose employed in the present study is several orders of magnitude higher than anticipated real-world dietary exposure. This gap is compounded by additional sources of uncertainty relevant to translation, including inter-individual variability in gastrointestinal absorption and hepatic metabolism, potential differences in oral bioavailability and first-pass metabolism between rodents and humans, and the possibility of cumulative exposure from concurrent consumption of multiple treated fruit sources over time, none of which were modeled in the present study. Taken together, while the supratherapeutic dose employed here was appropriate for establishing a proof-of-concept mechanistic framework, the substantial margin between our experimental exposure and both the regulatory ADI and estimated real-world dietary intake indicates that extrapolation to typical human dietary exposure scenarios should be made with caution.

In addition, atherosclerotic plaque characterization in this study relied on quantification of lesion area in the aortic root, a well-established and widely used index of atherosclerotic burden in apoE^−/−^ mice. However, plaque compositional features relevant to lesion vulnerability, including necrotic core size, collagen content, smooth muscle cell content, and quantitative macrophage burden, were not evaluated. As aortic tissue was preserved for protein extraction to enable histological and molecular analyses within the same animal cohort, consistent with the 3Rs principle of minimizing animal use, additional serial sections for comprehensive plaque phenotyping were not available. Consequently, our findings should be interpreted as reflecting overall lesion burden rather than plaque stability or vulnerability, and future studies incorporating detailed plaque compositional analysis are warranted to more fully characterize the impact of KT-30 on atherosclerotic plaque phenotype.

## 5. Conclusions

Using apoE^−/−^ mice to model human exposure to residual KT-30 on fruits, we demonstrate that 4-week KT-30 administration markedly elevates circulating cholesterol and inflammatory cytokine levels and accelerates atherosclerotic progression. KT-30 also disrupts hepatic lipid homeostasis, leading to increased hepatic cholesterol and cholesteryl ester levels and fatty acid accumulation. Mechanistically, KT-30 enhances de novo lipogenesis and cholesterol/triglyceride synthesis while reducing fatty acid uptake, lipoprotein uptake, and VLDL secretion. These metabolic disturbances, combined with impaired cholesterol efflux and heightened oxidative and inflammatory signaling, strongly suggest that KT-30 is associated with accelerated atherosclerosis progression in this model. As these findings are derived from a single genetically susceptible animal model exposed to a supratherapeutic dose over a relatively short (4-week) subacute period, without exposure biomonitoring or epidemiological corroboration, they should be regarded as hypothesis-generating rather than establishing definitive human cardiovascular risk. Nonetheless, these findings provide mechanistic evidence that KT-30 can promote pro-atherogenic alterations in a susceptible experimental model and underscore the need for future studies that incorporate environmentally relevant exposure levels, human biomonitoring, and epidemiological investigations before conclusions regarding human cardiovascular risk can be drawn.

## Data Availability

The datasets used and/or analyzed during the current study are available from the corresponding author upon reasonable request.

## Supplementary Materials

The following supporting information can be downloaded at: https://www.mdpi.com/article/10.3390/antiox15080953/s1, Table S1: The effects of KT-30 on mitochondrial dysfunction pathway-related proteins in the livers of apoE^−/−^ mice; Table S2: The effects of KT-30 on sirtuin signaling pathway-related proteins in the livers of apoE^−/−^ mice; Table S3: The effects of KT-30 on oxidative phosphorylation-related proteins in the livers of apoE^−/−^ mice; Table S4: The effects of KT-30 on integrin signaling-related proteins in the livers of apoE^−/−^ mice; Table S5: The effects of KT-30 on EIF2 signaling-related proteins in the livers of apoE^−/−^ mice; Table S6: The effects of KT-30 on glucocorticoid receptor signaling-related proteins in the livers of apoE^−/−^ mice; Table S7: The effects of KT-30 on acute phase response signaling-related proteins in the livers of apoE^−/−^ mice; Table S8: The effects of KT-30 on oncostatin M signaling-related proteins in the livers of apoE^−/−^ mice; Table S9: The effects of KT-30 on the regulation of eIF4 and p70S6K signaling-related proteins in the livers of apoE^−/−^ mice; Table S10: The effects of KT-30 on actin cytoskeleton signaling-related proteins in the livers of apoE^−/−^ mice.
