# Supplementary material for: The Plant Growth Regulator Forchlorfenuron (KT-30) Drives Atherosclerosis Progression via Lipid Homeostasis Disruption: Evidence from ApoE-Deficient Mice
Source: Antioxidants (Basel). 2026 Jul 30;15(8):953. doi: 10.3390/antiox15080953 (PMC13510049; doi:10.3390/antiox15080953)
Supplement: Supplementary file 1 [file antioxidants-15-00953-s001.zip › antioxidants-4446703-supplementary.pdf]

Table S1. The effects of KT-30 on mitochondrial dysfunction pathway-related proteins in the livers of apoE$^{-/-}$ mice

**Protein**	KT-30 vs. Vehicle	Fold change
**Amine oxidase [flavin-containing] A**	↑	>10
**NADH dehydrogenase [ubiquinone] 1 alpha subcomplex subunit 1**	↑	>10
**NADH dehydrogenase [ubiquinone] 1 alpha subcomplex subunit 5**	↑	>10
**Cytochrome c oxidase subunit 1**	↑	6.04
**Cytochrome c oxidase subunit NDUFA4**	↑	5.42
**NADH dehydrogenase [ubiquinone] iron-sulfur protein 6, mitochondrial**	↑	5.35
**NADH dehydrogenase [ubiquinone] 1 beta subcomplex subunit 7**	↑	3.2
**NADH dehydrogenase [ubiquinone] 1 beta subcomplex subunit 3**	↑	2.75
**NADH dehydrogenase [ubiquinone] 1 alpha subcomplex subunit 11**	↑	2.69
**Cytochrome b**	↑	2.49
**Mitochondrial fission 1 protein**	↑	2.46
**NADH-ubiquinone oxidoreductase chain 1**	↑	2.35
**NADH dehydrogenase [ubiquinone] 1 alpha subcomplex subunit 6**	↑	2.21
**Cytochrome c oxidase subunit 6B1**	↑	2.2
**ATP synthase-coupling factor 6, mitochondrial**	↑	2.14
**Succinate dehydrogenase cytochrome b560 subunit, mitochondrial**	↑	2.13
**Cytochrome c, somatic**	↑	2.11
**Peroxiredoxin-5, mitochondrial**	↑	2.1
**Cytochrome b-c1 complex subunit 8**	↑	2.1
**ATP synthase subunit delta, mitochondrial**	↑	2.05
**ATP synthase subunit gamma, mitochondrial**	↑	1.26
**ATP synthase F(0) complex subunit B1, mitochondrial**	-	-1.01
**Complex I intermediate-associated protein 30, mitochondrial**	↓	-2.53
**Mitogen-activated protein kinase 9**	↓	<-10

Table S2. The effects of KT-30 on sirtuin signaling pathway-related proteins in the livers of apoE$^{-/-}$ mice

Protein	KT-30 vs. Vehicle	Fold change
**NADH dehydrogenase [ubiquinone] 1 alpha subcomplex subunit 1**	↑	>10
**NADH dehydrogenase [ubiquinone] 1 alpha subcomplex subunit 5**	↑	>10
**[Pyruvate dehydrogenase (acetyl-transferring)] kinase isozyme 1, mitochondrial**	↑	>10
**ATP-dependent 6-phosphofructokinase, muscle type**	↑	>10
**Mitochondrial import inner membrane translocase subunit Tim9**	↑	>10
**Mitochondrial import inner membrane translocase subunit Tim13**	↑	6.92
**Cytochrome c oxidase subunit NDUFA4**	↑	5.42
**NADH dehydrogenase [ubiquinone] iron-sulfur protein 6, mitochondrial**	↑	5.35
**Phosphoenolpyruvate carboxykinase, cytosolic [GTP]**	↑	4.29
**Signal transducer and activator of transcription 3**	↑	3.45
**NADH dehydrogenase [ubiquinone] 1 beta subcomplex subunit 7**	↑	3.2
**Mitochondrial import receptor subunit TOM22 homolog**	↑	2.9
**NADH dehydrogenase [ubiquinone] 1 beta subcomplex subunit 3**	↑	2.75
**NADH dehydrogenase [ubiquinone] 1 alpha subcomplex subunit 11**	↑	2.69
**Cytochrome b**	↑	2.49
**NADH-ubiquinone oxidoreductase chain 1**	↑	2.35
**Mitochondrial import inner membrane translocase subunit Tim8 A**	↑	2.3
**NADH dehydrogenase [ubiquinone] 1 alpha subcomplex subunit 6**	↑	2.21
**Peptidyl-prolyl cis-trans isomerase F, mitochondrial**	↑	2.21
**Acetyl-coenzyme A synthetase, cytoplasmic**	↑	2.14
**ATP synthase-coupling factor 6, mitochondrial**	↑	2.14
**Succinate dehydrogenase cytochrome b560 subunit, mitochondrial**	↑	2.13
**ADP/ATP translocase 1**	↑	2.02
**ATP synthase F(0) complex subunit B1, mitochondrial**	-	-1.01
**Transcription intermediary factor 1-beta**	↓	-2
**Ubiquitin-like-conjugating enzyme ATG3**	↓	-2.17
**Complex I intermediate-associated protein 30, mitochondrial**	↓	-2.53
**Mitogen-activated protein kinase 3**	↓	-2.93
**Glycogen synthase kinase-3 beta**	↓	<-10

Table S3. The effects of KT-30 on oxidative phosphorylation-related proteins in the livers of apoE$^{-/-}$ mice

Protein	KT-30 vs. Vehicle	Fold change
**NADH dehydrogenase [ubiquinone] 1 alpha subcomplex subunit 1**	↑	>10
**NADH dehydrogenase [ubiquinone] 1 alpha subcomplex subunit 5**	↑	>10
**Cytochrome c oxidase subunit 1**	↑	6.04
**NADH dehydrogenase [ubiquinone] iron-sulfur protein 6, mitochondrial**	↑	5.35
**Cytochrome c oxidase subunit NDUFA4**	↑	5.42
**NADH dehydrogenase [ubiquinone] 1 beta subcomplex subunit 7**	↑	3.2
**NADH dehydrogenase [ubiquinone] 1 beta subcomplex subunit 3**	↑	2.75
**NADH dehydrogenase [ubiquinone] 1 alpha subcomplex subunit 11**	↑	2.69
**Cytochrome b**	↑	2.49
**NADH-ubiquinone oxidoreductase chain 1**	↑	2.35
**NADH dehydrogenase [ubiquinone] 1 alpha subcomplex subunit 6**	↑	2.21
**Cytochrome c oxidase subunit 6B1**	↑	2.2
**ATP synthase-coupling factor 6, mitochondrial**	↑	2.14
**Succinate dehydrogenase cytochrome b560 subunit, mitochondrial**	↑	2.13
**Cytochrome c, somatic**	↑	2.11
**Cytochrome b-c1 complex subunit 8**	↑	2.1
**ATP synthase subunit delta, mitochondrial**	↑	2.05
**ATP synthase subunit gamma, mitochondrial**	↑	1.26

Table S4. The effects of KT-30 on integrin signaling-related proteins in the livers of apoE$^{-/-}$ mice

Protein	KT-30 vs. Vehicle	Fold change
**Dual specificity mitogen-activated protein kinase kinase 2**	↑	>10
**Ras-related protein R-Ras**	↑	>10
**ADP-ribosylation factor 6**	↑	5.55
**Actin, alpha cardiac muscle 1**	↑	4.37
**ADP-ribosylation factor 4**	↑	2.36
**Gelsolin**	↑	2.26
**Profilin-1**	↑	2.23
**Myosin light chain kinase, smooth muscle**	↑	2.18
**GTPase HRas**	↓	-2.19
**Src substrate cortactin**	↓	-2.37
**Adapter molecule crk**	↓	-2.38
**Serine/threonine-protein kinase PAK 2**	↓	-2.4
**Growth factor receptor-bound protein 2**	↓	-2.5
**Mitogen-activated protein kinase 3**	↓	-2.93
**Phosphatidylinositol 3,4,5-trisphosphate 3-phosphatase and dual-specificity protein phosphatase PTEN**	↓	-3.24
**Glycogen synthase kinase-3 beta**	↓	<-10
**Growth factor receptor-bound protein 7**	↓	<-10
**Protein phosphatase 1 regulatory subunit 12A**	↓	<-10
**Ras-related C3 botulinum toxin substrate 2**	↓	<-10
**Rho-associated protein kinase 1**	↓	<-10

Table S5. The effects of KT-30 on EIF2 signaling-related proteins in the livers of apoE$^{-/-}$ mice

Protein	KT-30 vs. Vehicle	Fold change
**Dual specificity mitogen-activated protein kinase kinase 2**	↑	>10
**60S ribosomal protein L22-like 1**	↑	>10
**Protein argonaute-1**	↑	>10
**Ras-related protein R-Ras**	↑	>10
**40S ribosomal protein S21**	↑	6.92
**Serine/threonine-protein phosphatase PP1-gamma catalytic subunit**	↑	6.58
**Actin, alpha cardiac muscle 1**	↑	4.37
**Eukaryotic translation initiation factor 3 subunit M**	↑	3.58
**60S ribosomal protein L39**	↑	3.41
**60S acidic ribosomal protein P2**	↑	3.12
**Eukaryotic translation initiation factor 3 subunit H**	↑	3.07
**60S ribosomal protein L38**	↑	2.66
**40S ribosomal protein S15**	↑	2.4
**40S ribosomal protein S30**	↑	2.25
**60S ribosomal protein L37a**	↑	2.24
**40S ribosomal protein S29**	↑	2.18
**GTPase HRas**	↓	-2.19
**Growth factor receptor-bound protein 2**	↓	-2.5
**Mitogen-activated protein kinase 3**	↓	-2.93
**Glycogen synthase kinase-3 beta**	↓	<-10

Table S6. The effects of KT-30 on glucocorticoid receptor signaling-related proteins in the livers of apoE$^{-/-}$ mice

Protein	KT-30 vs. Vehicle	Fold change
**Dual specificity mitogen-activated protein kinase kinase 2**	↑	>10
**Ras-related protein R-Ras**	↑	>10
**Phosphoenolpyruvate carboxykinase, cytosolic [GTP]**	↑	4.29
**Signal transducer and activator of transcription 3**	↑	3.45
**Tyrosine aminotransferase**	↑	2.24
**Signal transducer and activator of transcription 1**	↓	-2
**Keratin, type II cytoskeletal 1b**	↓	-2.04
**Heat shock-related 70 kDa protein 2**	↓	-2.12
**DNA-directed RNA polymerase II subunit RPB2**	↓	-2.15
**GTPase HRas**	↓	-2.19
**Keratin, type I cytoskeletal 17**	↓	-2.36
**Signal transducer and activator of transcription 5B**	↓	-2.4
**Keratin, type I cytoskeletal 16**	↓	-2.41
**Growth factor receptor-bound protein 2**	↓	-2.5
**Keratin, type II cytoskeletal 6B**	↓	-2.55
**Mitogen-activated protein kinase 3**	↓	-2.93
**Mitogen-activated protein kinase 14**	↓	-2.96
**Keratin, type I cytoskeletal 14**	↓	-3.05
**Keratin, type II cytoskeletal 1**	↓	-3.11
**SWI/SNF complex subunit SMARCC2**	↓	-3.12
**Keratin, type I cytoskeletal 15**	↓	-3.21
**Keratin, type I cuticular Ha1**	↓	<-10
**Keratin, type II cytoskeletal 71**	↓	<-10
**Mitogen-activated protein kinase 9**	↓	<-10

Table S7. The effects of KT-30 on acute phase response signaling-related proteins in the livers of apoE$^{-/-}$ mice

Protein	KT-30 vs. Vehicle	Fold change
**Dual specificity mitogen-activated protein kinase kinase 2**	↑	>10
**Ras-related protein R-Ras**	↑	>10
**Interleukin-1 receptor accessory protein**	↑	>10
**Plasma kallikrein**	↑	>10
**Apolipoprotein A-I**	↑	3.85
**Signal transducer and activator of transcription 3**	↑	3.45
**Ferritin light chain 1**	↑	2.54
**Serum albumin**	↑	2.54
**Retinol-binding protein 4**	↑	2.44
**Alpha-1-antitrypsin 1-1**	↑	1.25
**GTPase HRas**	↓	-2.19
**Growth factor receptor-bound protein 2**	↓	-2.5
**Heme oxygenase 1**	↓	-2.54
**Mitogen-activated protein kinase 3**	↓	-2.93
**Mitogen-activated protein kinase 14**	↓	-2.96
**Mitogen-activated protein kinase 9**	↓	<-10

Table S8. The effects of KT-30 on oncostatin M signaling-related proteins in the livers of apoE$^{-/-}$ mice

Protein	KT-30 vs. Vehicle	Fold change
**Dual specificity mitogen-activated protein kinase kinase 2**	↑	>10
**Ras-related protein R-Ras**	↑	>10
**Signal transducer and activator of transcription 3**	↑	3.45
**Signal transducer and activator of transcription 1**	↓	-2
**GTPase HRas**	↓	-2.19
**Signal transducer and activator of transcription 5B**	↓	-2.4
**Growth factor receptor-bound protein 2**	↓	-2.5
**Mitogen-activated protein kinase 3**	↓	-2.93

Table S9. The effects of KT-30 on the regulation of eIF4 and p70S6K signaling-related proteins in the livers of apoE$^{-/-}$ mice

Protein	KT-30 vs. Vehicle	Fold change
**Protein argonaute-1**	↑	>10
**Dual specificity mitogen-activated protein kinase kinase 2**	↑	>10
**Ras-related protein R-Ras**	↑	>10
**Serine/threonine-protein phosphatase 2A activator**	↑	>10
**Serine/threonine-protein phosphatase 2A catalytic subunit alpha isoform**	↑	8.05
**40S ribosomal protein S21**	↑	6.92
**Eukaryotic translation initiation factor 3 subunit M**	↑	3.58
**Signal transducer and activator of transcription 3**	↑	3.45
**Eukaryotic translation initiation factor 3 subunit H**	↑	3.07
**40S ribosomal protein S15**	↑	2.4
**40S ribosomal protein S30**	↑	2.25
**40S ribosomal protein S29**	↑	2.18
**GTPase HRas**	↓	-2.19
**Growth factor receptor-bound protein 2**	↓	-2.5
**Mitogen-activated protein kinase 3**	↓	-2.93
**Mitogen-activated protein kinase 14**	↓	-2.96

Table S10. The effects of KT-30 on actin cytoskeleton signaling-related proteins in the livers of apoE$^{-/-}$ mice

Protein	KT-30 vs. Vehicle	Fold change
**Dual specificity mitogen-activated protein kinase kinase 2**	↑	>10
**Ras-related protein R-Ras**	↑	>10
**Myosin-10**	↑	5.08
**Actin, alpha cardiac muscle 1**	↑	4.37
**Gelsolin**	↑	2.62
**Profilin-1**	↑	2.23
**Myosin light chain kinase, smooth muscle**	↑	2.18
**GTPase HRas**	↓	-2.19
**Adapter molecule crk**	↓	-2.38
**Serine/threonine-protein kinase PAK 2**	↓	-2.4
**Growth factor receptor-bound protein 2**	↓	-2.5
**Mitogen-activated protein kinase 3**	↓	-2.93
**Ras GTPase-activating-like protein IQGAP1**	↓	-3.89
**Myosin-14**	↓	<-10
**Protein phosphatase 1 regulatory subunit 12A**	↓	<-10
**Ras-related C3 botulinum toxin substrate 2**	↓	<-10
**Rho-associated protein kinase 1**	↓	<-10
**Tyrosine-protein kinase CSK**	↓	<-10
